# Design and optimization of simplified inhibitors targeting *Escherichia coli* and *Klebsiella pneumoniae* IspE

**DOI:** 10.1039/d5md00874c

**Published:** 2026-01-08

**Authors:** Danica J. Walsh, Rawia Hamid, Tim Giele, Norbert Reiling, Matthias Rottmann, Mostafa M. Hamed, Anna K. H. Hirsch

**Affiliations:** a Helmholtz Institute for Pharmaceutical Research Saarland (HIPS) – Helmholtz Centre for Infection Research (HZI) Campus Building E8.1 66123 Saarbrücken Germany anna.hirsch@helmholtz-hips.de; b PharmaScienceHub Campus Building E 2.1 66123 Saarbrücken Germany; c Department of Pharmacy, Saarland University 66123 Saarbrücken Germany; d Microbial Interface Biology, Research Center Borstel, Leibniz Lung Center Parkallee 1 Borstel 23845 Germany; e German Center for Infection Research, DZIF Partner Site Hamburg-Lübeck-Borstel-Riems Borstel 23845 Germany; f Swiss Tropical and Public Health Institute, Medical Parasitology and Infection Biology Kreuzstrasse 2 CH-4123 Allschwil Switzerland; g University of Basel 4001 Basel Switzerland

## Abstract

The methylerythritol phosphate (MEP) pathway is essential for isoprenoid biosynthesis in many pathogenic bacteria but is absent in humans, making its enzymes attractive antibacterial targets. IspE catalyzes the ATP-dependent phosphorylation of 4-diphosphocytidyl-2-*C*-methylerythritol, a key step in this pathway. Using a previously identified optimized hit as a starting point, we designed and synthesized a focused library of twelve simplified analogues that retained essential pharmacophoric features while improving synthetic accessibility. Docking studies with *Escherichia coli* IspE guided the design and predicted binding orientations consistent with known ligand interactions. Biochemical evaluation of the library against *E. coli* and *Klebsiella pneumoniae* IspE revealed several low-micromolar inhibitors, confirming the predicted binding interactions. Structure–activity relationships indicated that the hydrophobic pocket adjacent to the cytidine-binding region is a key determinant of potency. Although the compounds showed limited whole-cell activity, these results demonstrate that simplified amide analogues can effectively engage the IspE active site and highlight the importance of the hydrophobic pocket in ligand binding. Overall, this work combines structure-based design, synthesis, and biochemical validation to provide a foundation for further optimization of simplified IspE inhibitors as potential antibacterial leads.

## Abstract 2

Simplified amide IspE inhibitors restore potency against *Klebsiella pneumoniae* and *Escherichia coli*, offering synthetically accessible antibacterial hits.

## Graphical abstract

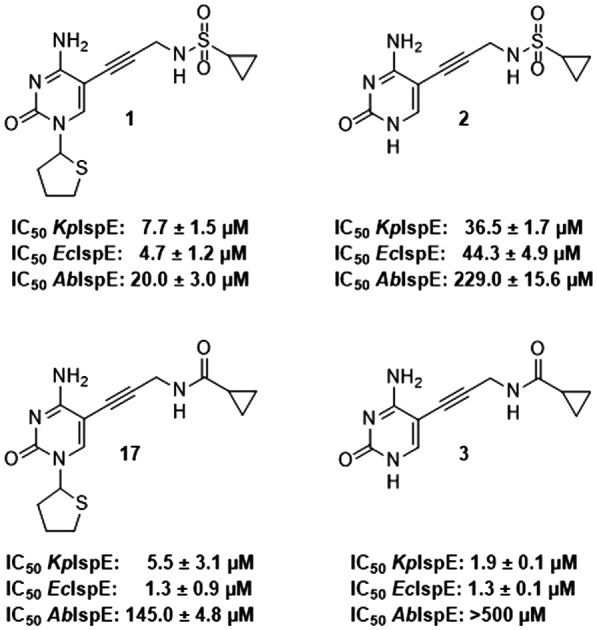

## Introduction

The rapid rise of antimicrobial resistance has resulted in a substantial decrease in the ability to treat infections and illnesses.^1^ This presents significant challenges in healthcare, food security, and global development.^2^ Established resistance is difficult to displace, while new resistance continues to emerge among diverse bacteria across the globe. There has been a sharp decline in new antibiotic development since the 1960s and an increase in the spread of resistance.^3^ Consequently, over a million lives are lost each year due to infections that can no longer be treated with existing antibiotics. As a result, there is an urgent need for the identification of new drug targets and inhibitors with unprecedented modes of action.

The 2-*C*-methyl-d-erythritol 4-phosphate (MEP) pathway is a metabolic pathway responsible for the biosynthesis of the universal isoprenoid precursors isopentenyl diphosphate (IDP) and dimethylallyl diphosphate (DMADP). Isoprenoids are vital to cell life as they are used in diverse processes such as hormone synthesis, cell–membrane maintenance, protein anchoring and *N*-glycosylation.^4^ The MEP pathway is essential for a wide range of clinically relevant pathogenic bacteria, such as *Klebsiella pneumoniae*, *Escherichia coli*, but is absent in humans.^5^ Although the MEP pathway features several viable drug targets, few inhibitors have been reported so far.^6^

**Fig. 1:**
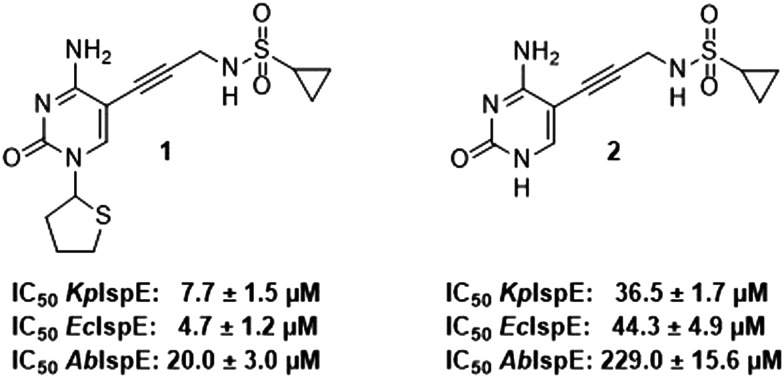
IC_50_ values for compounds 1 and 2 obtained from the biochemical assay with various IspE homologues.

Although IspE is highly conserved among Gram-negative pathogens, subtle amino acid differences exist between orthologs that can influence ligand binding and inhibitory activity. For instance, *K. pneumoniae* and *E. coli* IspE contain Pro182 and Cys211, whereas in *Acinetobacter baumannii* IspE these residues are replaced by Gln174 and Phe205, respectively. This substitution alters the local binding environment, meaning that different types of molecular interactions may occur at this site (Fig. 1).

**Fig. 2:**
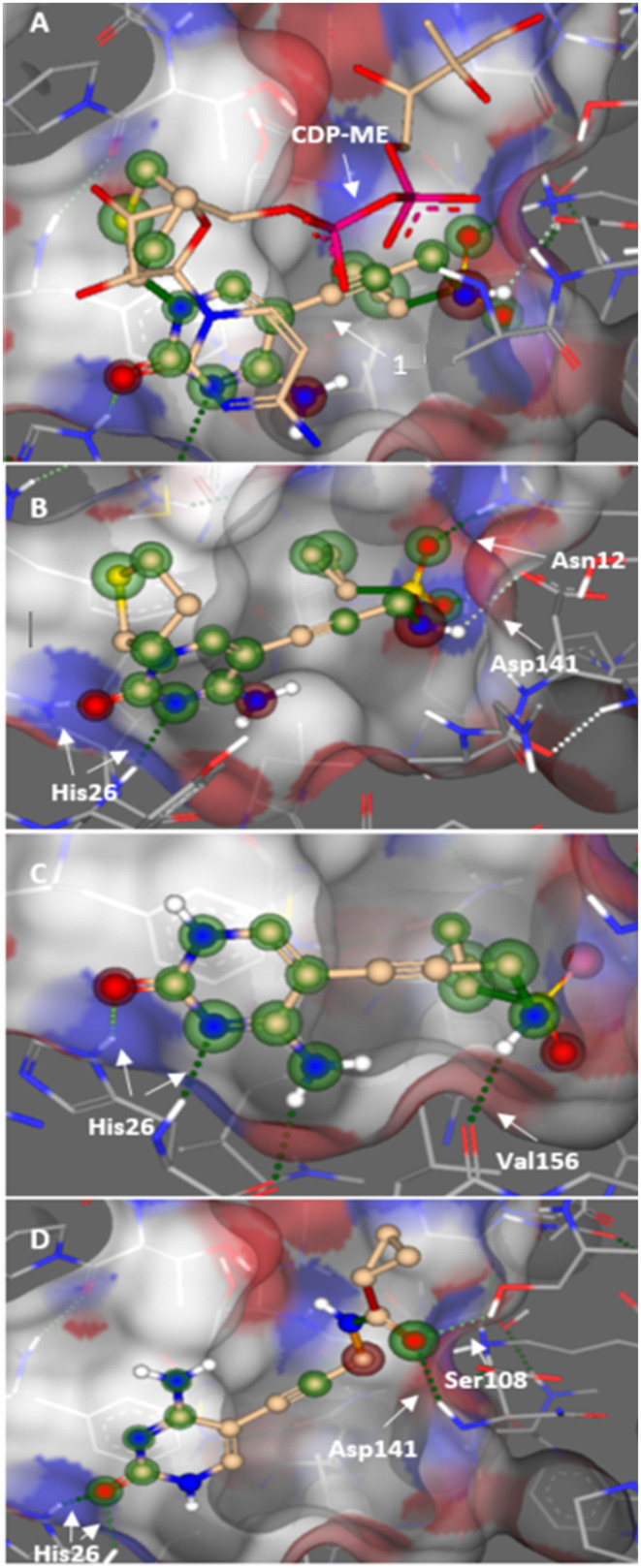
Overlay of docked pose of compound 1 and CDP–ME (A), compound 1 (B), compound 2 (C), and compound 3 (D) in *Escherichia coli* IspE (PDB ID: 1OJ4). Green spheres indicate favorable contributions to the binding affinity, while red spheres indicate unfavorable contributions as calculated by the HYDE scoring function in SeeSAR. The size of each sphere reflects the magnitude of the interaction.

Previously, we identified and developed inhibitor 1, which demonstrated activity towards *K. pneumoniae* IspE (*Kp*IspE), *E. coli* IspE (*Ec*IspE) and *Acinetobacter baumannii* IspE (*Ab*IspE).^7–9^ In our previous reports, it was shown that this inhibitor occupies the substrate binding site and a small, hydrophobic sub-pocket at the active site of IspE where CDP–ME binds, bypassing the ATP binding site (Fig. 2A). In the most recent study, we attempted to simplify inhibitor 1, by removal of the tetrahydrothiophene ring (2) (Fig. 1). This was done in an effort to simplify the synthesis of this class of inhibitors by eliminating a low-yielding and time-consuming step. While this ultimately decreased potency, compound 2 maintained significant activity towards both *Kp*IspE and *Ec*IspE.

In this study, we aimed to optimize activity, while maintaining the simplified structure. This was done through a docking study that led to the synthesis of a library of amide inhibitors (Table 1). Based on a docking study using *Ec*IspE (PDB ID: 1OJ4), we predicted that the simplified amide derivatives would be accommodated within the substrate pocket in a manner similar to compounds 1 and 2 (Fig. 2). After our initial modeling was completed, we deposited high-resolution crystal structures of *E. coli* IspE in complex with ligands (PDB IDs **8QCC**, **8QCN**).^9^ We therefore compared our docking poses to these X-ray crystal structures and found the key binding features to be conserved: the cytidine/cytosine group occupies the same general region and engages the same nearby residues (including His26 and interactions consistent with Asn12/Asp141), and the hydrophobic sub-pocket that accommodates the cyclopentyl/cyclopropyl moieties in our models is present in the crystal structures. These comparisons validate that the docking models used to design the amide library captured the principal features of ligand binding observed in the experimental structures. In Fig. 2, it can be observed that the cytosine group of compounds 1 (B), 2 (C) and 3 (D) interact with His26, while the cyclopropyl moiety is in a slightly different position in 2 and 3. The sulfonamide group of compound 1 hydrogen bonds with Asn12 and Asp141, while 2 interacts with Val156 and the carbonyl group of 3 with Asp141 and Ser108. These predicted contacts correspond well to those observed in the deposited *E. coli* IspE crystal structures (PDB IDs **8QCC** and **8QCN**) where the cytidine/cytosine fragment occupies the same pocket and establishes comparable polar contacts.

**Table 1:** IC_50_ values obtained from the biochemical assay against *Klebsiella pneumoniae* and *Escherichia coli* IspE for amide derivatives 3–10

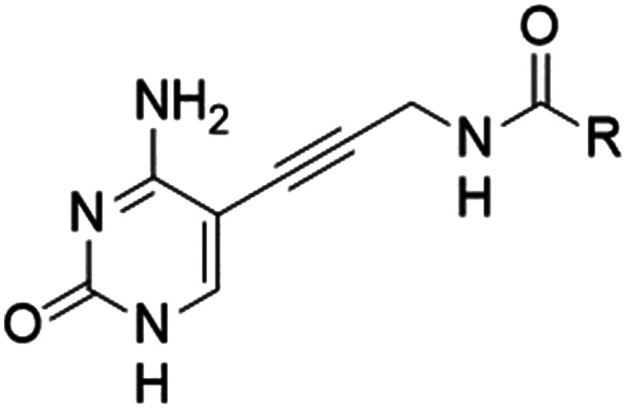			
Inhibitor	R group	IC_50_*Kp*IspE (μM)	IC_50_*Ec*IspE (μM)
3	Cyclopropyl	1.9 ± 0.1	1.3 ± 0.1
4	Methyl	18 ± 4.4	8.5 ± 4.3
5	Ethyl	1.3 ± 0.1	1.3 ± 0.1
6	*n*-Bu	11.6 ± 1.8	5.0 ± 1.5
7	*t*-Bu	7.5 ± 1.5	1.3 ± 0.1
8	Cyclohexyl	19.4 ± 1.5	14.8 ± 1.2
9	–CH=CH_2_	23.3 ± 6.9	5.9 ± 1.1
10	–CH_2_CF_3_	4.6 ± 0.5	4.5 ± 1.8

**Scheme 1:**
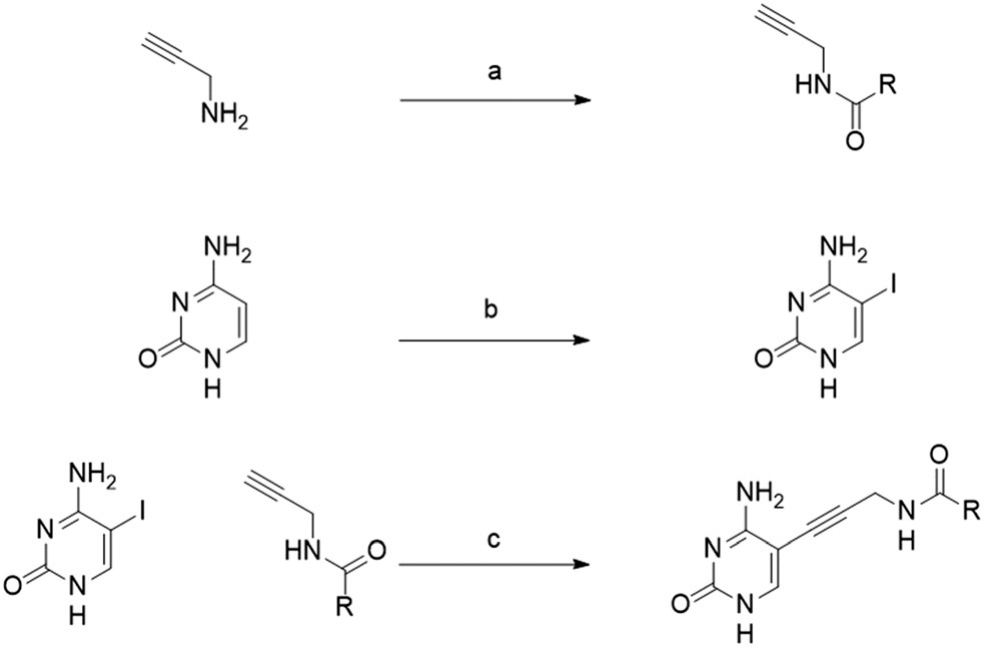
General synthesis for amide derivatives. a) TEA, DCM, 0 °C – R.T., 30 min b) I_2_, HIO_3_, AcOH, 40 °C, 12 c) [PdCl_2_(PPh_3_)_2_], CuI, TEA, DMF, r.t., 12 h

The synthesis was carried out in three steps (Scheme 1). First, propargylamine was reacted with the appropriate acid chloride. Iodo-cytosine was synthesized by reacting iodic acid with iodine in acetic acid at room temperature overnight. Final products were obtained *via* a Sonogashira cross-coupling reaction, involving a terminal acetylene and aryl or alkenyl halides in the presence of Pd(0) and copper(i) catalysts with base.

## Results

First, we synthesized compound 3, which retains the cyclopropyl group present in compounds 1 and 2 on the amide linker. All compounds were evaluated for inhibitory activity (S1.1). This compound showed a significant boost in activity compared to compound 2 towards both *Kp*IspE and *Ec*IspE with an IC_50_ of 1.9 ± 0.1 μM and 4.7 ± 1.2 μM, respectively. Although all activity towards *Ab*IspE was lost, we focused on *Kp* and *Ec*IspE here due to the similarities in the substrate binding pocket.^10^ It has been shown that *Ec*IspE and *Kp*IspE possess an 82% sequence identity, with conserved residues, which are involved in binding to ATP and CDP–ME.^9^ By contrast, *Ab*IspE shares only 39% sequence identity with *Ec*IspE and 42% with *Kp*IspE. Subsequently, we synthesized a library of nine amide derivatives to target *Ec* and *Kp*IspE in order to establish a structure–activity relationship (SAR) that would help to optimize this novel library of inhibitors.

Several other aliphatic chains or rings, including methyl (4), ethyl (5), *n*-butyl (6), *t*-butyl (7), vinyl (9) and cyclohexyl (8) were evaluated. The ethyl group had comparable activity as the cyclopropyl against *Kp*IspE and improved activity against *Ec*IspE. Based on this observation, we synthesized the methyl (4) and *n*-butyl (6) derivatives but, neither compound was as active as the ethyl derivative (5). The larger cyclohexyl group (8) also showed a decrease in activity compared to 3. Although these compounds showed activity towards *Kp*IspE and *Ec*IpsE, they lost activity towards *Ab*IspE at concentrations up to 500 μM. Compounds 3 and 5 emerged as the most active against *Kp*IspE and 7 and 5 against *Ec*IspE. To further evaluate this simplified class of derivatives, we synthesized compounds 11–15 (Table 2). In derivatives 11 and 12, we omitted the amide group and a tertiary amine was employed instead. For compound 13 we maintained the amide and removing the carbonyl of the cytosine ring, while in 14 and 15 the linker placement was modified. In compound 15, the linker was also modified to make it more flexible by eliminating the triple bond. In compound 16, the alkyne was also reduced, but with the linker in the original position.

**Table 2:** IC_50_ values obtained from the biochemical assay against *Klebsiella pneumoniae* and *Escherichia coli* IspE for the second set of derivatives 11–16

Compound	Structure	IC_50_*Kp*IspE (μM)	IC_50_*Ec*IspE (μM)
11	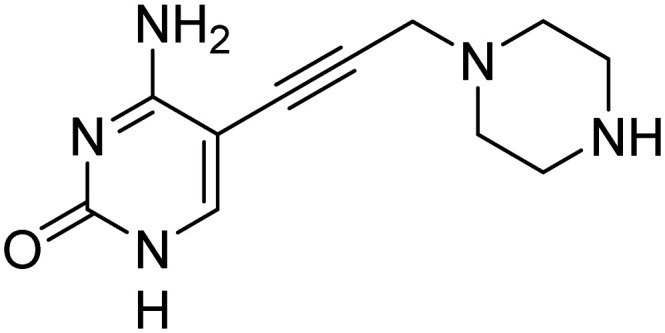	>500	90.2 ± 67.5
12	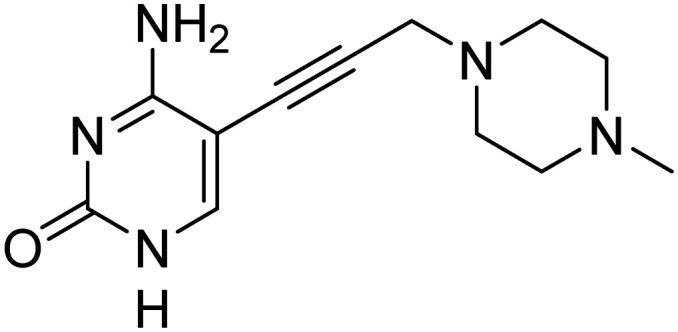	>500	>500
13	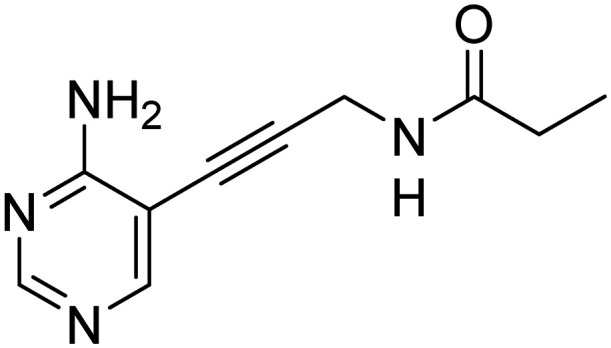	>500	>500
14	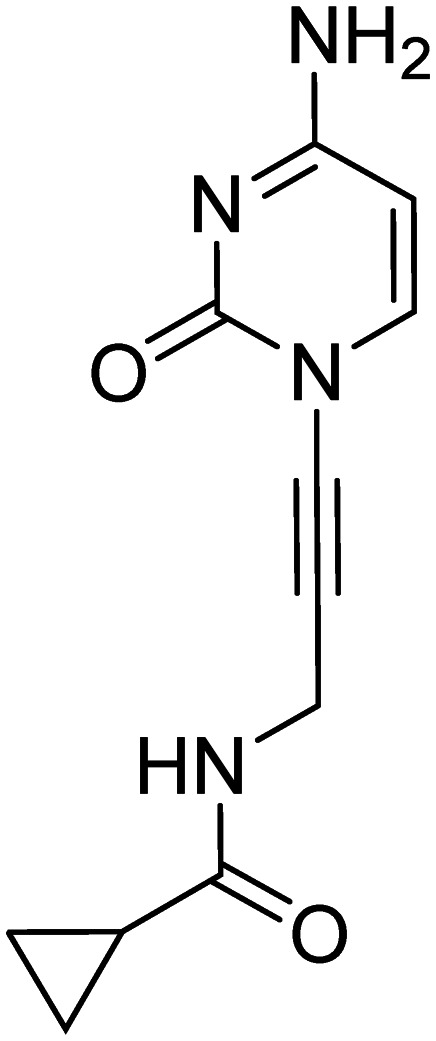	N/A^a^	N/A^a^
15	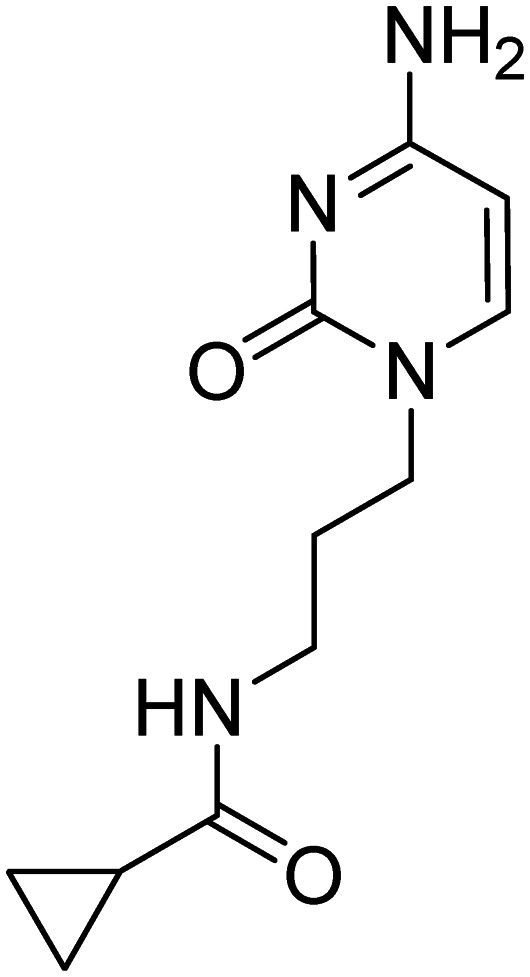	>500	>500
16	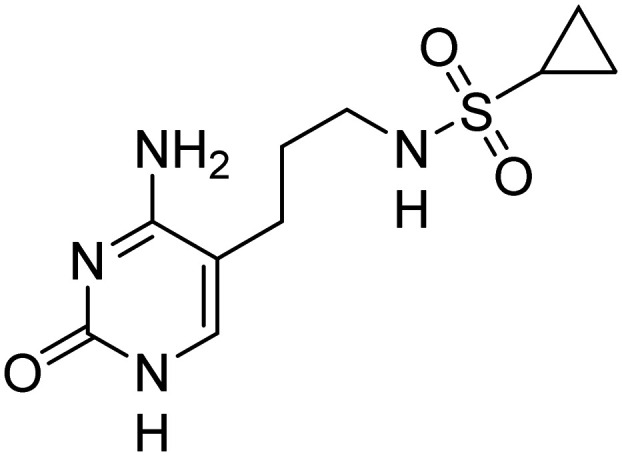	>500	>500

a N/A denotes that we were unable to evaluate the compound due to poor solubility in the enzyme assay.

The tertiary amine 11 showed a significant decrease in activity towards *Ec*IspE and a complete loss of activity towards *Kp*IspE at 500 μM. Next, we synthesized compound 13 to assess the importance of the carbonyl group on the aryl ring, while maintaining aromaticity leading to a complete loss of activity. Additionally, we synthesized compounds 14 and 15 to determine how the placement of the alkyne moiety would affect activity. Unfortunately, 14 was only slightly soluble in DMSO and insoluble in the 5% DMSO buffer solution used for IC_50_ assays, hindering its biological evaluation. Compound 15, which lacks the alkyne and has an alkane linker, also showed a complete loss of activity. Lastly, compound 16 was synthesized to determine if the placement of the saturated linker was the major reason for loss of activity. No activity was observed in compound 16. In conclusion, we have assessed that the placement and the rigidity of the linker as well as the presence of the amide, are important for the activity.

**Fig. 3:**
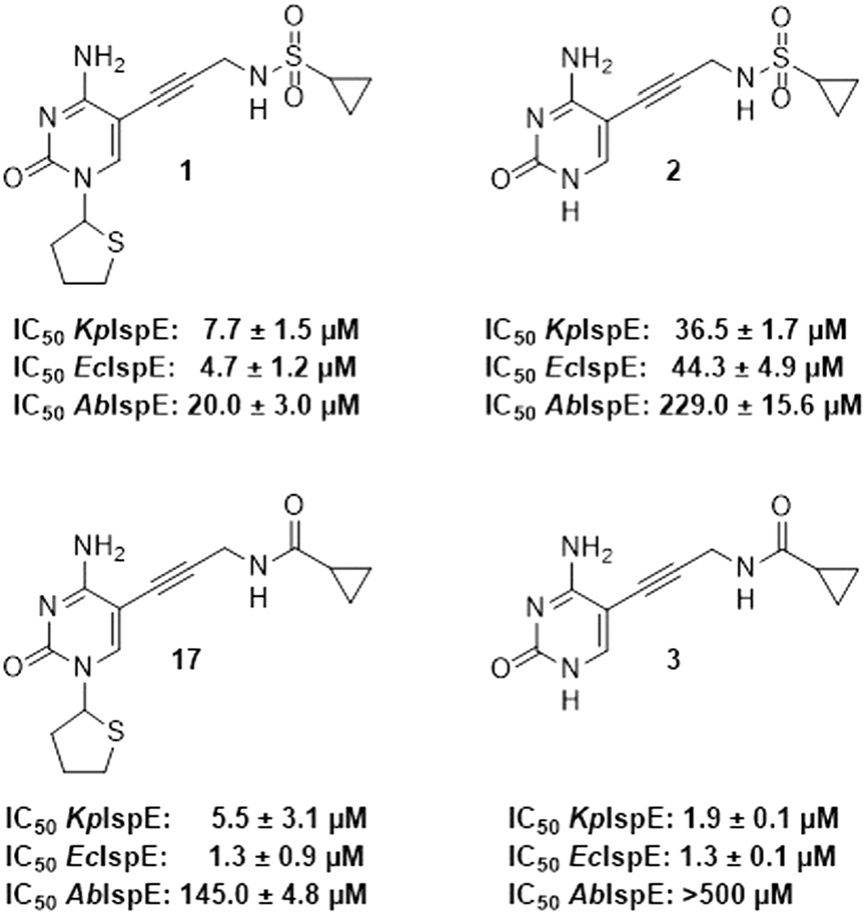
IC_50_ values for compounds 1, 2, 3 and 17 obtained from the biochemical assay with various IspE homologues.

As a next step, the tetrahydrothiophenyl moiety was reinstated to give compound 17 (Fig. 3), aiming to restore the potency lost upon its removal in compound 2. Since removal of this group from 1 to 2 resulted in decreased activity across all orthologs, we anticipated that reinstating it would enhance potency for *Ec*IspE and *Kp*IspE as well. However, compound 17 displayed activity comparable to 3 against *Ec*IspE and a slight decrease against *Kp*IspE, while showing improved inhibition of *Ab*IspE. This unexpected trend suggests that the tetrahydrothiophenyl group contributes differently depending on the overall binding orientation of the scaffold. The results may indicate that compound 3, or its derivatives, adopt a slightly altered binding mode compared to the parent sulfonamide 1, which could explain why reinstating the tetrahydrothiophenyl ring did not yield the anticipated potency increase. Therefore, while the data confirm that this group can modulate activity, they do not conclusively demonstrate that it is more critical for *Ab*IspE than for *Ec*IspE or *Kp*IspE. Further structural or co-crystallization studies will be required to clarify these binding-mode differences.

We determined the dissociation constant (*K*_d_) for six selected compounds for the target protein *Kp*IspE (Table 3). *K*_d_ values were assessed using microscale thermophoresis (MST) (S1.2) and IC_50_ values were determined using the enzymatic assay as described (S1.1). As expected, the *K*_d_ values found were in line with their IC_50_ values. Compound 8 was chosen because of its relatively lower activity to serve as a comparison.

**Table 3:** *K*_d_ values and IC_50_ values for selected compounds obtained from microscale thermophoresis and the biochemical assay, respectively, with *Klebsiella pneumoniae* IspE

Compound	*K*_d_ *Kp*IspE	IC_50_*Kp*IspE
1	7.7 ± 1.5 μM	3.5 ± 0.2 μM
3	2.0 ± 0.2 μM	1.9 ± 0.1 μM
5	1.1 ± 0.1 μM	1.3 ± 0.1 μM
8	30.0 ± 7.2 μM	19.4 ± 1.5 μM
17	8.4 ± 0.5 μM	5.5 ± 3.1 μM

To further evaluate these compounds as early drug candidates, StarDrop (version: 7.0.1.29911) was used to predict parameters in Lipinski's rule of 5 such as number of rotatable bonds, number of hydrogen bond donors (HBD), number of hydrogen bond acceptors, clog *P*, and polar surface area (PSA, Table 4).

**Table 4:** StarDrop predicted values for clog *P*, PSA and most basic p*K*_a_ as well as the number of rotatable bonds, hydrogen bond donating and accepting groups and IC_50_ values from the biochemical assay

Compound	IC_50_*Kp*IspE (μM)	log *D*	log *P*	Rotatable bonds	HBD	HBA	PSA	Most basic p*K*_a_
1	7.7 ± 1.5	0.09669	1.004	6	2	7	107.1	6.5
2	36.5 ± 1.7	−1.026	−0.3395	5	3	7	117.9	6.6
3	1.9 ± 0.1	−0.3387	−0.2886	5	3	6	100.9	6.2
4	18 ± 4.4	−0.9024	−0.6713	4	3	6	100.9	6.4
5	1.3 ± 0.1	−0.5209	−0.2316	5	3	6	100.9	6.4
6	11.6 ± 1.8	0.02716	0.3068	7	3	6	100.9	6.5
7	7.5 ± 1.5	0.3219	0.2242	5	3	6	100.9	6.1
8	19.4 ± 1.5	0.3153	0.5164	5	3	6	100.9	6.5
9	23.3 ± 6.9	−0.8195	−0.5079	5	3	6	100.9	6.3
10	4.6 ± 0.5	0.427	0.8066	6	3	6	100.9	5.9
11	>500	−1.079	−0.7783	3	3	6	87.04	9.6
12	>500	−0.6512	−0.4294	3	2	6	78.25	9.0
13	>500	−0.6978	1.275	5	2	5	80.9	4.6
14	N/A	−0.3724	−0.1803	5	2	6	90.01	5.2
15	>500	−0.2503	−0.4501	6	2	6	90.01	6.1
16	>500	−0.832	−0.4789	6	3	7	117.9	7.3
17	5.5 ± 3.1	0.6873	0.9077	6	2	6	90.01	6.3

All active compounds conformed to the rule of 5, having fewer than 5 HBD, fewer than 10 HBA, a molecular weight under 500 Dalton, and a clog *P* of under 5.

We also evaluated the *in vitro* ADMET (absorption, distribution, metabolism, excretion, and toxicity) properties (Table 5). Optimizing ADMET properties is important in the drug-design and development process to create safer and more efficacious drug candidates and enable a true multiparameter optimization. This evaluation also helps to explain how pharmacokinetic processes happen. Compounds 3, 17, and 2 were chosen. Compound 3 was selected because it was among the most active and has a close structural resemblance to the simplified sulfonate 2. Compound 17 was selected to directly compare the sulfonamide 1 to the amide derivative.

**Table 5:** *In vitro* ADMET profiling (kinetic solubility, toxicity, mouse plasma, mouse liver S9 and mouse liver microsome) results for selected compounds

	Kinetic solubility		Log *D*_7.4_	Toxicity	Mouse plasma	Mouse liver S9	Mouse liver microsomes
Compound	Mean (μM)	SD		HepG2	*t*_1/2_ [min]	*t*_1/2_ [min]/Cl_int_ [μL mg^−1^ min^−1^]	*t*_1/2_ [min]/Cl_int_ [μL mg^−1^ min^−1^]
1	>1000	0	0.34	<10% at 100 μM	>240	>120/<5.8	>120/<11.6
2	520.1	13.57	−1.41	<10% at 100 μM	>240	>120/<5.8	>120/<11.6
3	115.1	55.29	−1.62	<10% at 100 μM	>240	>120/<5.8	>120/<11.6
17	206.3	140.3	0.14	<10% at 100 μM	>240	>120/<5.8	>120/<11.6

We observed that 1 has a higher solubility compared to its simplified structure 2. When the sulfonamide is replaced with an amide, we observed a decrease in solubility. Like the sulfonamides 1 and 2, the evaluated amides showed no cytotoxicity towards HepG2 cells and were stable in mouse liver S9 fraction, liver microsomes and plasma (Table 5).

Next, we evaluated the same four compounds towards the wild type *E. coli* strain *K12* (S1). Since these compounds showed no activity, they were then evaluated for inhibitory activity towards *E. coli delta-acrB*. An important factor in drug resistance are mechanisms that actively remove antibiotics from the inside of the cell, such as efflux pumps. The a*crB* strain was selected to assess, if this compound class lacks antibacterial activity due to efflux in *E. coli*. None of the five selected compounds demonstrated inhibitory activity towards *E. coli delta-acrB* (Table S1), suggesting that permeability is the main bottleneck.

Next, several compounds were selected for evaluation towards *Mycobacterium tuberculosis* and *Plasmodium falciparum* because IspE inhibitors have been previously studied on these organisms as well (Fig. S1).^11^ Unfortunately, the compounds showed no antimicrobial activity towards either pathogen.

## Conclusion

Our study aimed to restore potency towards *Ec* and *Kp*IspE of the previously simplified derivatives of IspE inhibitors. To achieve this, we kept the simplified scaffold and through a docking–guided study developed an amide derivative library. We were able to restore potency towards *Ec* and *Kp*IspE for multiple compounds and towards *Ab*IspE for compound 17. Our findings provide a synthetically simple but potent IspE inhibitors. Although it was found that this class of compounds lacks inhibitory activity in whole-cells assays, there is still potential for augmentation in order to gain activity. We note that co-crystal structures of *E. coli* IspE with ligands have now been deposited (PDB IDs **8QCC**, **8QCN**), which already provide high-resolution structural context for ligand binding and confirm the presence of the hydrophobic subpocket exploited by our designs.

Thus, the next step in the project is the elucidation of co-crystal structures with 2 and 3 in order to gain a better understanding of their binding mode, and to conduct further experiments to confirm the reason for the lack of antibacterial activity.

## Author contributions

Synthesis was performed by D. J. W. and T. G. Biological evaluation of compounds was performed by R. H., M. R., N. R. and D. J. W. D. J. W. performed the MST assays for the determination of *K*_d_ values. D. J. W. performed the docking studies. M. M. H. and A. K. H. H. designed and coordinated the study, which was supervised by A. K. H. H. D. J. W. wrote the manuscript and M. M. H. and A. K. H. H. proofread it, with the contribution of all authors. All authors have given approval of the final version.

## Conflicts of interest

There are no conflicts to declare.

## Supplementary Material

MD-OLF-D5MD00874C-s001

## Data Availability

All data supporting the findings of this study are available within the article and its supplementary information (SI) files. Supplementary information is available. See DOI: https://doi.org/10.1039/d5md00874c.
