# Supplementary material for: Design and optimization of simplified inhibitors targeting Escherichia coli and Klebsiella pneumoniae IspE
Source: RSC Med Chem. 2026 Jan 8;17(3):1687–93. doi: 10.1039/d5md00874c (PMC12902931; doi:10.1039/d5md00874c)
Supplement: MD-017-D5MD00874C-s001 [file MD-017-D5MD00874C-s001.pdf]

Supplementary Information (SI) for RSC Medicinal Chemistry.
This journal is © The Royal Society of Chemistry 2025

Design and Optimization of Simplified Inhibitors Targeting *E. coli* and *K. pneumoniae* IspE

Danica J. Walsh[1,2], Rawia Hamid[1,2,3], Tim Giele[1,2], Norbert Reiling[5], Mostafa M. Hamed[1], Matthias Rottmann[6], Mostafa M. Hamed[1,2], Anna K.H. Hirsch[1,2,3*]

1. Helmholtz Institute for Pharmaceutical Research Saarland (HIPS) – Helmholtz Centre for Infection Research (HZI), Campus Building E8.1, 66123 Saarbrücken, Germany.

2. PharmaScienceHub, 66123 Saarbrücken, Germany

3. Saarland University, Department of Pharmacy, 66123 Saarbrücken, Germany.

4. Hamburg School of Food Science, Institute of Food Chemistry, Grindelallee 117, 20146 Hamburg, Germany

5. The German Center for Infection Research, Research Center Borstel, Leibniz Lung Center, Parkallee 22, D-23845 Borstel, Germany

6. Swiss Tropical and Public Health Institute, Medical Parasitology and Infection Biology, Kreuzstrasse 2, CH-4123 Allschwil, Switzerland

*Correspondence e-mail: anna.hirsch@helmholtz-hips.deanna.hirsch@helmholtz-hips.de

Contents

Experimental 2
1. Biology 2
1.1 *Kp*IspE IC_{50} assay 2
1.2 Microscale thermophoresis for K_d determination: 2
1.3 Mininal inhibitory concentration (MIC) determinations. 2
1.4 Mycobacterium tuberculosis growth analysis in liquid culture: 3
1.5 Cytotoxicity Assay 4
1.6 Solubility Determination (Kinetic Turbidimetric Solubility) 5
2. Molecular docking of Amide Series 5
3. Chemistry 5
3.1 Materials and Methods 5
3.2 General information 6
3.3 Synthetic Strategy 6
3.4 Experimental Procedures 7
3.5 Spectra: 11

Experimental

1. Biology

1.1 *Kp*IspE IC_{50} assay

Assays were conducted in 384-well plates (Corning) with a transparent flat bottom. Assay buffer A (total volume, 30 µL) contained 200 mM Tris hydrochloride, pH 7.6, (containing 20 mM KCl, 10 mM MgCl2, 10 mM DTT), 2 mM NADH, 2 mM phosphoenolpyruvic acid, 0.2 U of pyruvate kinase (PK), 0.2 U of lactate dehydrogenase (LDH) and 0.3 µM IspE K. pneumonia. Assay buffer B contained 200 mM Tris hydrochloride, pH 7.6, 2 mM ATP and 0.6 mM CDP-ME. Compound stocks were made in DMSO (20mM) and further diluted in Tris HCl buffer to a concentration of 1.3 mM in 96 well plates, so that the starting concentration in the assay is 120 µM. Dilution series were performed with dilution step 1:2 and approximately covered the concentration range of 120 µM to 0.23 µM in the assay. The impact of the tested compounds on auxiliary enzymes in the photometric activity inhibition assay was tested as follows. Well 1 will serve as a blank and well 3 as a positive control. Assay buffer A (30 µL) was transferred into wells 1, 3, 5, 7... etc. Compound (6 µL) was added to wells 5, 7, 9, 11… etc. Tris HCl (30 µL) was transferred into well 1, while buffer B (30 µL) was transferred into wells 3, 5, 7, 9… etc, to start the reaction. The 384 well plate was centrifuged for 1 min. The reactions were monitored photometrically (room temperature) at 340 nm in a plate reader (SpectraMax5, Molecular Dynamics). Initial rate values were evaluated with a nonlinear regression method using the program Dynafit.[1] CDP-ME was used as starting substrate, and was synthesized and purified as previously described.[2] KpIspE was obtained and purified as previously reported.[3]

1.2 Microscale thermophoresis for K_d determination:

The microscale thermophoresis (MST) (Serial no. 201709-BR-N024, Monolith NT.115 Micro Scale Thermophoresis, NanoTemper Technologies GmbH.) was performed according to the standard protocol from the manufacturer NanoTemper Technologies GmbH using the Monolith His-Tag Labeling Kit RED-tris-NTA 2nd Generation kit. The buffer used was HEPES (50 mM), MgCl2 (5 mM) and Tween (0.05%) at pH 7.6 for all other compounds. The protein concentration of 50 nM was used and the ligand was tested at the highest soluble concentration, which was 2 mM for most of the compounds under the assay conditions. A 1:1 dilution of the ligand over 16 samples was performed using a stock of ligand stock (in water) diluted in HEPES buffer. Non-hydrophobic capillary tubes were used. A pretest to check for the labelling and compound fluorescence was performed before every sample, followed by a binding affinity (Kd) determination. Each sample was measured after 15 min incubation time at RT and analyzed in MO Control version 1.6.

1.3 Mininal inhibitory concentration (MIC) determinations.

As start OD_{600} of *Escherichia coli* strains K12 and Δ*acrB* (efflux pump mutant JW0451-2) as well as*Klebsiella pneumoniae* (DSM30104/ATCC13883) we used 0.03 in a total volume of 200 µL in lysogeny broth (LB) containing the compounds predissolved in DMSO (max. 1%). Final compound concentrations prepared from serial dilutions ranged from 0.02 to 100 µg/mL (double values for each concentration) depending on their antibacterial activity. The ODs were determined after addition of the compounds and again after incubation for 18 h at 37 °C and 50 rpm in 96 well plates (Sarstedt, Nümbrecht, Germany) using a FLUOStar Omega (BMG labtech, Ortenberg, Germany). Given MIC values are means of two independent determinations and are defined as the lowest concentration of compounds that reduced OD_{600} by ≥ 95%. To obtain percent inhibition values for a given sample, the ODs were compared to those of DMSO controls.

Table S1. Percent inhibition of select compounds against K. pneumoniae, E. coli K12 and E. coli delta-acrB.

	inhibition of *K. pneumoniae*	inhibition of *E. coli K12*	inhibition of *E. coli delta-acrB*

**1**	<10% at 100 µM	<10% at 100 µM	<10% at 100 µM
**2**	<10% at 100 µM	<10% at 100 µM	<10% at 100 µM
**3**	<10% at 100 µM	<10% at 100 µM	<10% at 100 µM
**17**	<10% at 100 µM	<10% at 100 µM	<10% at 100 µM

1.4 Mycobacterium tuberculosis growth analysis in liquid culture:

Mycobacterium tuberculosis H37Rv (ATCC 27294) was cultured in 7H9 complete medium (BD Difco; Becton Dickinson) supplemented with oleic acid-albumin-dextrose-catalase (OADC, 10%; BD), 0.2% glycerol and 0.05% Tween80, as previously described. At mid-log phase (OD600 = 0.4), the cultures were harvested and frozen in aliquots at −80 °C (PMID: 28249101). Frozen aliquots were thawed and centrifuged (3700× g, 10 min). Supernatants were discarded and bacteria were thoroughly resuspended (10 times) in 7H9 medium (10% OADC) in the absence of glycerol and Tween80 by use of a syringe and a 26-gauge syringe needle. **Compound efficacy was assessed using a broth microdilution assay, based on a previously described protocol (PMID: 34235672) with a few modifications.** Briefly, twofold serial dilutions of each compound were prepared starting at a concentration of 64 µM. Triplicate wells were inoculated with *M. tuberculosis* at 1×10^5 CFU per well in a final volume of 100 µL, using clear, U-bottom 96-well microtiter plates to evaluate anti-tubercular activity. Plates were left to incubate at 37 °C, in ziplock bags within humidified sealed boxes for 7 days, prior to visual examination of bacterial growth using an inverted mirror device. 30 ul of a 0.02 % (w/v) aqueous solution of resazurin (Cayman Chemical, Ann Arbor, USA) was then added to each well and the plates left to incubate at 37 C for 4h. Reduction of resazurin to resorufin, indicative of cell viability, was measured with a fluorescence plate reader (Synergy2, Agilent) at wavelengths 540 (ex)/ 590(em) nm. Fluorescence values were converted to percentage growth relative to DMSO treated cells (100 % growth). Final DMSO concentrations did not exceed 1 % (v/v). The obtained values were normalized to fluorescence values of the solvent control (DMSO)-treated bacteria set to 100%), and MIC95 of each compound was determined. MIC95 was defined as the minimum concentration of the compound required to achieve a reduction in fluorescence by 95%.

Figure S1. Summary of activity against Mycobacterium tuberculosis H37Rv (ATCC 27294).

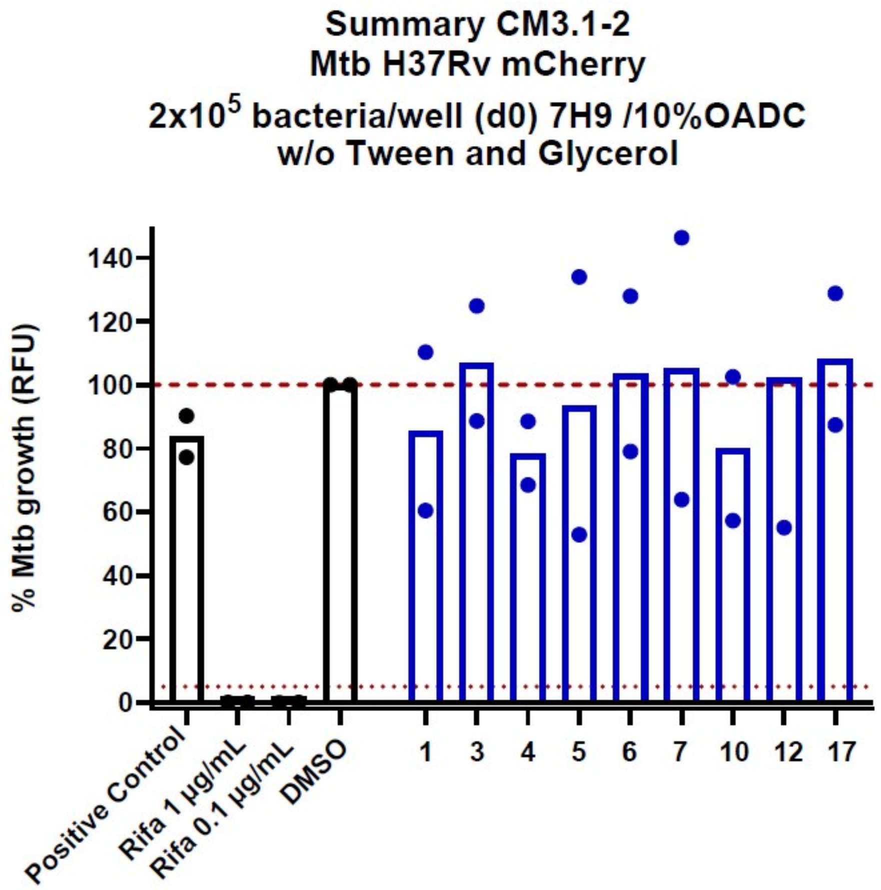

1.5 Cytotoxicity Assay

To obtain information regarding the toxicity of our compounds, their impact on the viability of human cells was investigated. HepG2 cells (2×10^4 cells per well) were seeded in 96-well, flat-bottomed culture plates in 100 µL culture medium (DMEM containing 10% fetal calve serum, 1% penicillin-streptomycin). Twenty-four hours after seeding the cells, medium was removed and replaced by medium containing test compounds in a final DMSO concentration of 1%. Compounds were tested in duplicates at a single concentration or, for CC_{50} determination, at 8 concentrations that were prepared *via* 2-fold serial dilutions in 1% DMSO/medium. Epirubicin and doxorubicin were used as positive controls in serial dilutions starting from 10 µM, and rifampicin was used as a negative control (at 100 µM). The living cell mass was determined 48 h after treatment with compounds by adding 0.1 volumes of 3-(4,5-dimethylthiazol-2-yl)-2,5-diphenyltetrazolium bromide (MTT) solution (5 mg/mL sterile PBS) (Sigma, St. Louis, MO) to the wells. After incubating the cells for 30 min at 37 °C (atmosphere containing 5% CO_2), medium was removed and MTT crystals were dissolved in 75 µL of a solution containing 10% SDS and 0.5% acetic acid in DMSO. The optical density (OD) of the samples was determined photometrically at 570 nm in a PHERAstar Omega plate reader (BMG labtech, Ortenberg, Germany). To obtain percent viability for each sample, their ODs were related to those of DMSO controls. At least two independent measurements were performed

for each compound. The calculation of CC_{50} was performed using the nonlinear regression function of GraphPad Prism 10 (GraphPad Software, San Diego, CA, USA).

1.6 Solubility Determination (Kinetic Turbidimetric Solubility)

The desired compounds were sequentially diluted in DMSO in a 96-well plate. 7.5 µL of each well were transferred into another 96-well plate and mixed with 142.5 µL of PBS. Plates were shaken for 5 min at 600 rpm at room temperature (r.t.), and the absorbance at 620 nm was measured. Absorbance values were normalized by blank subtraction and plotted using GraphPad Prism 8.4.2 (GraphPad Software, San Diego, CA, USA). Solubility (S) was determined based on the First X value of AUC function using a threshold of 0.005.

1.7 Kinetic Turbidimetric Solubility

The desired compounds were sequentially diluted in DMSO in a 96-well plate. 1.5 µL of each well were transferred into another 96-well plate and mixed with 148.5 µL of PBS pH 7.4 to reach 1% DMSO/PBS.. Plates were shaken for 5 min at 600 rpm at room temperature, and the absorbance at 620 nm was measured. Absorbance values were normalized by blank subtraction and plotted using GraphPad Prism 10 (GraphPad Software, San Diego, CA, USA). Solubility (S) was determined based on the First X value of AUC function using a threshold of 0.005.

1.8 Lipophilicity Determination

$LogD_{7.4}$ was analyzed using an HPLC−based method. The UV retention time of reference compounds with known $LogD_{7.4}$ was determined and plotted toward their $LogD_{7.4}$. Linear regression was used to determine the $LogD_{7.4}$ of unknown compounds. Analysis was performed using a Vanquish Flex HPLC system with variable wavelength detector (Thermo Fisher, Dreieich, Germany) with the following conditions: EC150/2 NUCLEODUR C18 Pyramid column, 5 µM (Macherey Nagel, Düren, Germany); eluent A: 50 mM NH_4OAc pH 7.4, eluent B: acetonitrile, and flow: 0.6 mL/min. The gradient was set to 0−100% B from 0 to 2.5 min, 100% B from 2.5 to 3.0 min, 100−0% B from 3.0 to 3.2 min, and 0% B from 3.2−5.0.

1.9 Metabolic Stability in Liver S9 Fractions

For the evaluation of combined phase I and phase II metabolic stability, the compound (1 µM) was incubated with 1 mg/mL pooled mouse liver S9 fraction (Xenotech, Kansas City, USA), 2 mM NADPH, 1 mM UDPGA, 10 mM $MgCl_2$, 5 mM GSH and 0.1 mM PAPS at 37 °C for 120 min. The metabolic stability of testosterone, verapamil and ketoconazole were determined in parallel to confirm the enzymatic activity of mouse S9 fractions. The incubation was stopped after defined time points by precipitation of aliquots of S9 enzymes with 2 volumes of cold internal standard solution (15 nM diphenhydramine in 10% methanol/acetonitrile). Samples were stored on ice until the end of the incubation and precipitated protein was removed by centrifugation (15 min, 4 °C, 4,000 g). The remaining test compound at the different time points was analyzed by HPLC-MS/MS (Vanquish Flex coupled to a TSQ Altis Plus, Thermo Fisher, Dreieich, Germany) and used to determine half-life ($t_{1/2}$).

1.10 Metabolic Stability in Mouse Liver Microsomes

For the evaluation of phase I metabolic stability, the compound (1 µM) was incubated with 0.5 mg/mL pooled mouse liver microsomes (Xenotech, Kansas City, USA), 2 mM NADPH, 10 mM $MgCl_2$ at 37 °C for 120 min on a microplate shaker (Eppendorf, Hamburg, Germany). The metabolic stability of

testosterone, verapamil and ketoconazole was determined in parallel to confirm the enzymatic activity of mouse liver microsomes. The incubation was stopped after defined time points by precipitation of aliquots of enzymes with 2 volumes of cold internal standard solution (15 nM diphenhydramine in 10% methanol/acetonitrile). Samples were stored on ice until the end of the incubation and precipitated protein was removed by centrifugation (15 min, 4 °C, 4,000 g). The remaining test compound at the different time points was analyzed by HPLC-MS/MS (Vanquish Flex coupled to a TSQ Altis Plus, Thermo Fisher, Dreieich, Germany) and used to determine half-life ($t_{1/2}$).

1.11 Stability in Plasma

To determine stability in plasma, the compound (1 µM) was incubated with pooled CD-1 mouse plasma (Neo Biotech, Nanterre, France). Samples were taken at defined time points by mixing aliquots with 4 volumes of ice-cold internal standard solution (12.5 nM diphenhydramine in 10% methanol/acetonitrile). Samples were stored on ice until the end of the incubation and precipitated protein was removed by centrifugation (15 min, 4 °C, 4,000 g, 2 centrifugation steps). The remaining test compound at the different time points was analyzed by HPLC-MS/MS (Vanquish Flex coupled to a TSQ Altis Plus, Thermo Fisher, Dreieich, Germany). The plasma stability of procain, propantheline and diltiazem were determined in parallel to confirm the enzymatic activity.

1.12 Cytotoxicity assay

To obtain information regarding the toxicity of our compounds, their impact on the viability of human cells was investigated. HepG2 cells ($2x10^4$ cells per well) were seeded in 96-well, flat-bottomed culture plates in 100 µL culture medium (DMEM containing 10% fetal calve serum, 1% penicillin-streptomycin). Twenty-four hours after seeding the cells, medium was removed and replaced by medium containing test compounds in a final DMSO concentration of 1%. Compounds were tested in duplicates at a single concentration or, for CC_{50} determination, at 8 concentrations that were prepared *via* 2-fold serial dilutions in 1% DMSO/medium. Epirubicin and doxorubicin were used as positive controls in serial dilutions starting from 10 µM, and rifampicin was used as a negative control (at 100 µM). The living cell mass was determined 48 h after treatment with compounds by adding 0.1 volumes of 3-(4,5-dimethylthiazol-2-yl)-2,5-diphenyltetrazolium bromide (MTT) solution (5 mg/mL sterile PBS) (Sigma, St. Louis, MO) to the wells. After incubating the cells for 30 min at 37 °C (atmosphere containing 5% CO_2), medium was removed and MTT crystals were dissolved in 75 µL of a solution containing 10% SDS and 0.5% acetic acid in DMSO. The optical density (OD) of the samples was determined photometrically at 570 nm in a PHERAstar Omega plate reader (BMG labtech, Ortenberg, Germany). To obtain percent viability for each sample, their ODs were related to those of DMSO controls. At least two independent measurements were performed for each compound. The calculation of CC_{50} was performed using the nonlinear regression function of GraphPad Prism 10 (GraphPad Software, San Diego, CA, USA).

2. Molecular docking of Amide Series

Molecular docking studies were performed using SeeSAR version 13.1 (SeeSAR V13.1. www.biosolveit.de/SeeSAR (accessed 2025-01-18)). The crystal structure of the target protein in complex with CDP-ME (PDB ID: 1OJ4) was imported directly into SeeSAR without further modification. The binding site was defined by selecting the co-crystallized CDP-ME ligand, which automatically delineated the binding pocket for subsequent docking calculations. The amide series compounds were then loaded into the docking module and subjected to molecular docking using standard parameters, generating 10 poses per ligand with standard

clash tolerance and default ring conformation tolerance settings. Following pose generation, all docking solutions were rescored using the HYDE scoring function. For each pose, intramolecular and intermolecular clash energies were computed alongside analysis of the torsional strain profile to evaluate the geometric quality of the docked conformations. The highest- ranked pose according to the HYDE score was selected as the representative binding mode for each compound. These top-ranked poses were subsequently subjected to detailed manual inspection to confirm the preservation of key interactions observed between the cytosine moiety of CDP-ME and the binding site, while simultaneously evaluating the distinct interaction patterns and binding contributions of the novel amide substituents introduced in the designed ligand series. Compounds that satisfied these important interactions were further prioritized by HYDE score.

3. Chemistry

3.1 Materials and Methods

All reagents used for chemical synthesis were purchased from commercial suppliers, and used without further purification. All chemical yields refer to purified compounds and were not optimized. Reaction progress was monitored using TLC silica gel 60 F_{254} aluminum sheets, and visualization was accomplished by UV at 254 nm. Column chromatography was performed using the automated flash chromatography system CombiFlash® Rf (Teledyne Isco) equipped with RediSepRf silica columns. Preparative RP-HPLC was was perofmed using an UltiMate 3000 Semi-Preparative System (Thermo Fisher Scientific) with nucleodur® C18 Gravity (250 mm × 10 mm, 5 µm) column. ^{1}H and ^{13}C NMR spectra were recorded as indicated on a Bruker Avance Neo 500 MHz (^{1}H, 500 MHz; ^{13}C, 126 MHz) with prodigy cryoprobe system or a Bruker Fourier 300 (^{1}H, 300 MHz; 13C, 75 MHz) instrument. Chemical shifts were recorded as δ values in ppm units and referenced against the residual solvent peak ($CDCl_3$, δ = 7.26, 77.16; DMSO-d_6, δ = 2.50, 39.52, MeOH-d_4: δ = 3.35, 4.78, 49.3). Splitting patterns describe apparent multiplicities and are designated as s (singlet), br s (broad singlet), d (doublet), dd (doublet of doublet), t (triplet), q (quartet), m (multiplet). Coupling constants (*J*) are given in hertz (Hz). Low resolution mass analytics and purity control of final compounds was carried out either using an Ultimate 3000-MSQ LCMS system (Thermo Fisher Scientific) consisting of a pump, an autosampler, MWD detector and a ESI quadrupole mass spectrometer. Purity of all compounds used in biological assays was ≥ 95%. High resolution mass spectra were recorded on a ThermoFisher Scientific (TF, Dreieich, Germany) Q Exactive Focus system equipped with heated electrospray ionization (HESI)-II source. Final products were dried at high vacuum.

3.2 General information

Chemical and Analytical Methods.

^{1}H- and ^{13}C-NMR spectra were recorded as indicated on a Bruker Avance Neo 500 MHz with prodigy cryoprobe system or a Bruker Fourier 300 instrument. Chemical shifts are given in parts per million (ppm), and referenced against the residual solvent peak. Coupling constants (J) are given in Hertz. Low-resolution mass analytics and purity control of final compounds were carried out either using an Ultimate 3000-MSQ LCMS system (Thermo Fisher Scientific)**,** consisting of a pump, an autosampler, MWD detector and an ESI quadrupole mass spectrometer. High resolution mass spectra were recorded on a ThermoFisher Scientific (TF, Dreieich, Germany) Q Exactive Focus system equipped with heated electrospray ionization (HESI)-II source. Reagents were used as obtained from commercial suppliers without further purification. Procedures were not optimized regarding yield. Column chromatography was performed using the automated flash chromatography system CombiFlash® Rf (Teledyne Isco) equipped with RediSepRf silica columns. Preparative HPLC was performed using an UltiMate 3000 Semi-Preparative System (Thermo Fisher Scientific) with nucleodur® C18 Gravity (250 mm × 10 mm, 5 µm) column. Final products were dried at high vacuum.

General Procedure A for the formation of an amide:

The acyl chloride (1 eq.) was added dropwise to a solution of propargyl amine (1 eq.) and Et3N (1.1 eq.) in dry CH2Cl2, at 0 °C. The mixture was left to stir at 25 °C for 30 min and concentrated in vacuo. Products were purified via column chromatography.

General procedure B for the Sonogashira cross coupling:

To an Ar-charged flask charged with iodocytosine (1.0 eq.), the previously synthesized alkyne (2 eq.), and Et3N (3.0 eq.) in anhydrous DMF, [PdCl2(PPh3)2] (0.1 eq.) and CuI (0.2 eq.) were added at 25 °C. The mixture was left to stir at 25 °C for 12 h. Water was added to the flask and the reaction mixture filtered. The solid filtrate was collected and purified via HPLC.

General procedure C for the Sonogashira cross coupling:

To an Ar-charged flask charged with iodocytosine (1.0 eq.), the previously synthesized alkyne (2 eq.), and Et3N (3.0 eq.) in anhydrous THF, [PdCl2(PPh3)2] (0.1 eq.) and CuI (0.2 eq.) were added, and the reaction heated to 65°C. The mixture was left to stir at 65°C for 4 h. Water was added to the flask and the reaction mixture filtered. The solid filtrate was collected and purified via HPLC.

3.3 Synthetic Strategy

Synthesis of compounds **3 - 17** and their intermediates was achieved *via* the route depicted in Scheme S1.

Scheme S1. General scheme for the synthesis

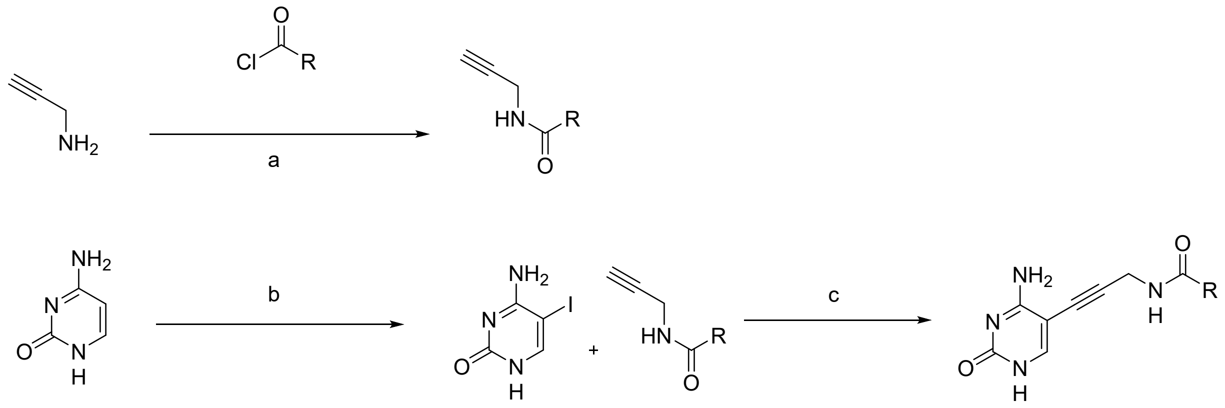

Reagents and condition: a) TEA, DCM, 0°C-r.t, 30min; b) I_2, HIO_3, AcOH, 40°C, 12h; c) [PdCl2(PPh3)2], CuI, TEA, DMF, r.t, 12 h.

Preparative HPLC Purifications

Purifications *via* preparative RP-HPLC were carried out using two possible conditions: *Condition A*: gradient 5–100% CH3CN + 0.05% HCOOH in water + 0.05% HCOOH in 53 min at a flow rate of 5 mL/min. The sample was dissolved in DMSO and manually injected to the HPLC system. *Condition B*: gradient 20–100% CH3CN + 0.05% HCOOH in water + 0.05% HCOOH in 35 min at a flow rate of 5 mL/min. The sample was dissolved in DMSO and manually injected to the HPLC system.

3.4 Experimental Procedures

Synthesis of N-(3-(4-amino-2-oxo-1,2-dihydropyrimidin-5-yl)prop-2-yn-1-yl)cyclopropanecarboxamide (3)

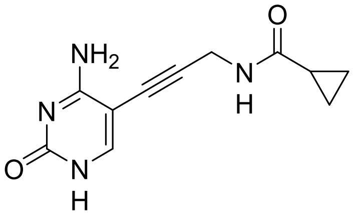

Compound **3** was synthesized according to the general method B. The crude product was purified by preparative HPLC (Condition A). **3** was obtained as a white solid, (73 mg, 0.3 mmol, 23%). ^{1}H NMR (500 MHz, DMSO-d$_6$) δ 7.67 (s, 1H), 4.04 (s, 2H), 2.66-2.61 (m, 1H), 0.97-0.95 (m, 4H). ^{13}C NMR (126 MHz, DMSO-d$_6$) δ 177.14, 155.40, 150.95, 136.22, 129.64, 85.37, 82.71, 32.81, 15.69, 9.37. Purity via HPLC: 100%; MS (ESI+) *m/z* 233 [*M*+H]$_+$; HRMS (ESI+) *m/z* calcd for $C_{11}H_{12}N_4O_2$ [*M*+H]+: 233.0960 found 233.1031.

N-(3-(4-amino-2-oxo-1,2-dihydropyrimidin-5-yl)prop-2-yn-1-yl)acetamide (4)

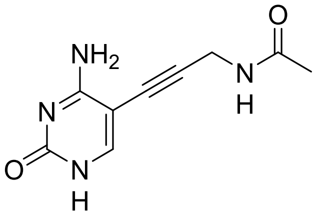

Compound **4** was synthesized according to the general method B. The crude product was purified by preparative HPLC (Condition A). **4** was obtained as a light green solid, (54 mg, 0.26 mmol, 32%). ^{1}H NMR (500 MHz, DMSO-d$_6$) δ 8.30 (s, 1H), 4.07 (d, *J*= 3.9 Hz, 1H), 1.83 (s, 3H). ^{13}C NMR (126 MHz, DMSO-d$_6$) δ 169.52, 158.12, 151.24, 141.64, 129.59, 85.97, 81.62, 29.66, 22.89. Purity via HPLC: 100%; MS (ESI+) *m/z* 207 [*M*+H]$_+$;HRMS (ESI+) *m/z* calcd for $C_9H_{10}N_4O_2$ [*M*+H]+: 207.0804 found 207.0872.

Synthesis of N-(3-(4-amino-2-oxo-1,2-dihydropyrimidin-5-yl)prop-2-yn-1-yl)propionamide (5)

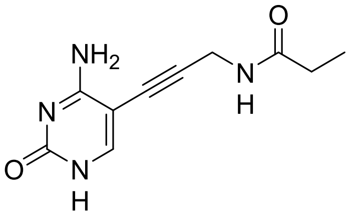

Compound **5** was synthesized according to the general method B. The crude product was purified by preparative HPLC (Condition A). **5** was obtained as a white solid, (133 mg, 0.6 mmol, 18%). ^{1}H NMR (500 MHz, DMSO-d$_6$) δ 8.52 (s, 1H), 4.16 (s, 2H), 1.59-1.54 (m, 1H), 0.69-0.68 (m, 4H). ^{13}C NMR (126 MHz, DMSO-d$_6$) δ 173.30, 158.02, 154.61, 140.16, 130.50, 90.14, 88.82, 34.36, 30.64, 7.60. Purity via HPLC: 100%; MS (ESI+) *m/z* 221 [*M*+H]$_+$; HRMS (ESI+) *m/z* calcd for $C_{10}H_{16}N_4O_2$ [*M*+H]+: 221.0960 found 221.1032.

Synthesis of N-(3-(4-amino-2-oxo-1,2-dihydropyrimidin-5-yl)prop-2-yn-1-yl)pentanamide (6)

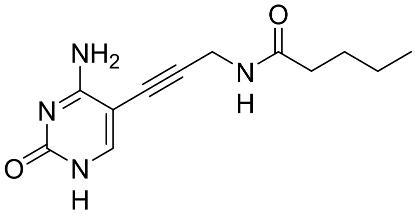

Compound **6** was synthesized according to the general method B. The crude product was purified by preparative HPLC (Condition A). **6** was obtained as a white solid, (71 mg, 0.29 mmol, 32%). ^{1}H NMR (500 MHz, DMSO-d$_6$) δ 8.25 (s, 1H), 4.09-4.06 (m, 2H), 2.10 (t, *J*= 7.3 Hz, 2H), 1.49 (tt, *J*= 7.5, 7.5 Hz, 2H), 1.27 (tq, *J*= 7.5, 7.4 Hz, 2H), 0.87 (t, *J*= 7.3 Hz, 3H). ^{13}C NMR (126 MHz, DMSO-d$_6$) δ 172.54, 150.26,

141.32, 135.65, 122.72, 91.08, 88.73, 35.34, 29.60,27.76, 22.26, 14.18. Purity via HPLC: 100%; MS (ESI+) *m/z* 249 [*M*+H]$_{+}$; HRMS (ESI+) *m/z* calcd for $C_{12}H_{16}N_4O_2$ [*M*+H]+: 249.1273 found 249.1199.

Synthesis of N-(3-(4-amino-2-oxo-1,2-dihydropyrimidin-5-yl)prop-2-yn-1-yl)pivalamide (7)

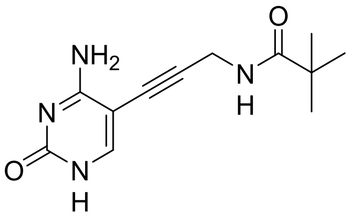

Compound **7** was synthesized according to the general method B. The crude product was purified by preparative HPLC (Condition A). **7** was obtained as a white solid, (38 mg, 0.15 mmol, 32%). ^{1}H NMR (500 MHz, DMSO-d$_6$) δ 7.63 (s, 1H), 4.06 (s, 1H), 1.11 (s, 9H). ^{13}C NMR (126 MHz, DMSO-d$_6$) δ 177.91, 167.59, 163.05, 155.54, 140.16, 93.18, 87.08, 38.53, 30.01, 27.75. Purity via HPLC: 100%; MS (ESI+) *m/z* 249 [*M*+H]$_{+}$; HRMS (ESI+) *m/z* calcd for $C_{12}H_{16}N_4O_2$ [*M*+H]+: 249.1273 found 249.1345.

N-(3-(4-amino-2-oxo-1,2-dihydropyrimidin-5-yl)prop-2-yn-1-yl)cyclohexanecarboxamide (8)

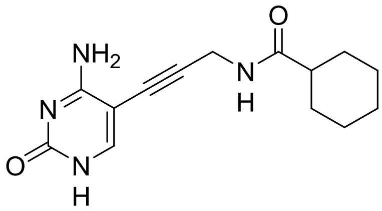

Compound **8** was synthesized according to the general method C. The crude product was purified by preparative HPLC (Condition A). **8** was obtained as a white solid. (35 mg, 0.13 mmol, 15%). ^{1}H NMR (500 MHz, DMSO-d$_6$) δ 7.66 (s, 1H), 3.83 (s, 2H), 2.14-2.08 (m, 1H), 1.71-1.60 (m, 4H), 1.36-1.10 (m, 6H). ^{13}C NMR (126 MHz, DMSO-d$_6$) δ 173.55, 165.87, 150.12, 147.48, 120.43, 84.38, 82.12, 45.62, 42.54, 28.86, 24.86, 22.11. Purity via HPLC: 100%; MS (ESI+) *m/z* 275 [*M*+H]+; HRMS (ESI+) *m/z* calcd for $C_{14}H_{18}N_4O_2$ [*M*+H]$_{+}$: 275.1430 found 274.1494.

N-(3-(4-amino-2-oxo-1,2-dihydropyrimidin-5-yl)prop-2-yn-1-yl)acrylamide (9)

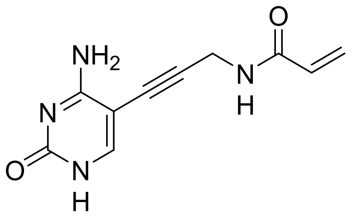

Compound **9** was synthesized according to the general method B. The crude product was purified by preparative HPLC (Condition A). **9** was obtained as a white solid, (24mg, 0.11 mmol, 16%). ^{1}H NMR (500 MHz, DMSO-d$_6$) δ 8.56 (s, 1H), 6.54 (dd, *J*=10.1, 15.0 Hz, 1H), 6.14 (dd, *J*= 17.1, 1.9 Hz, 1H), 5.15 (dd, *J*= 7.9, 2.2 Hz, 1H), 3.86 (s, 2H). ^{13}C NMR (126 MHz, DMSO-d$_6$) δ 164.90, 155.46, 150.08, 147.13, 132.60, 129.09, 126.44, 93.08, 80.16, 29.68. Purity via HPLC: 100%; MS (ESI+) *m/z* 219 [*M*+H]+; HRMS (ESI+) *m/z* calcd for $C_{10}H_{10}N_4O_2$ [*M*+H]$_{+}$: 219.0804 found 219.0873.

Synthesis of N-(3-(4-amino-2-oxo-1,2-dihydropyrimidin-5-yl)prop-2-yn-1-yl)-3,3,3-trifluoropropanamide (10)

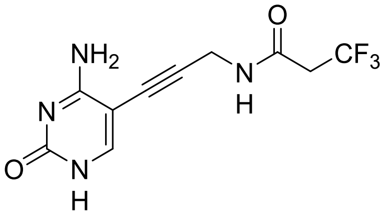

Compound **10** was synthesized according to the general method B. The crude product was purified by preparative HPLC (Condition A). **10** was obtained as a white solid, (47 mg, 0.17 mmol, 29%) ^{1}H NMR (500 MHz, DMSO-d_6) δ 7.67 (s, 1H), 4.15 (s, 1H), 3.30 (s, 1H). ^{13}C NMR (126 MHz, DMSO-d_6) δ 163.00, 157.39, 146.65, 141.63, 126.34, 124.14, 91.67, 89.29, 33.93, 29.93. ^{19}F NMR (500 MHz, $CDCl_3$) δ -62.93. Purity via HPLC: 100%; MS (ESI+) *m/z* 275 [*M*+H]$_+$; HRMS (ESI+) *m/z* calcd for $C_{10}H_9F_3N_4O_2$ [*M*+H]+: 275.0678 found 275.0748.

4-amino-5-(3-(piperazin-1-yl)prop-1-yn-1-yl)pyrimidin-2(1H)-one (11)

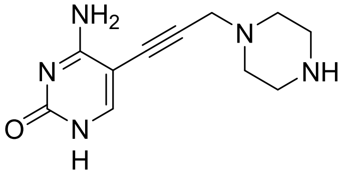

Compound **11** was synthesized according to the general method B. The crude product was purified by preparative HPLC (Condition A). **11** was obtained as a white solid, (42 mg, 0.18 mmol, 18%). ^{1}H NMR (500 MHz, DMSO-d_6) δ 7.58 (s, 1H), 3.55 (s, 2H), 2.76 (m, 4H), 2.35 (m, 4H). ^{13}C NMR (126 MHz, DMSO-d_6) δ 165.98, 164.36, 154.12, 146.76, 88.39, 76.96, 75.35, 70.02, 53.76, 49.06, 48.53, 45.79, 45.65. Purity via HPLC: 100%; MS (ESI+) *m/z* 233 [*M*+H]$_+$; HRMS (ESI+) *m/z* calcd for $C_{11}H_{15}N_5O$ [*M*+H]+: 233.1277 found 233.0984.

4-amino-5-(3-(4-methylpiperazin-1-yl)prop-1-yn-1-yl)pyrimidin-2(1H)-one (12)

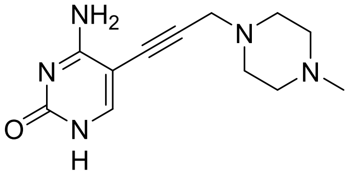

Compound **12** was synthesized according to the general method B. The crude product was purified by preparative HPLC (Condition A). **12** was obtained as a white solid, (37 mg, 0.15 mmol, 17%). ^{1}H NMR (500 MHz, DMSO-d_6) δ 7,70 (s, 1H), 3.59 (s, 2H), 2.76-2.75 (m, 8H), 1.78 (s, 3H). ^{13}C NMR (126 MHz, DMSO-d_6) δ 165.68, 163.52, 155.45, 147.17, 88.45, 77.04, 74.77, 69.80, 53.06, 48.61, 46.17, 45.71, 42.81. Purity via HPLC: 100%; MS (ESI+) *m/z* 248 [*M*+H]$_+$; HRMS (ESI+) *m/z* calcd for $C_{12}H_{17}N_5O$ [*M*+H]+: 248.1501 found 248.1433.

N-(3-(4-aminopyrimidin-5-yl)prop-2-yn-1-yl)propionamide (13)

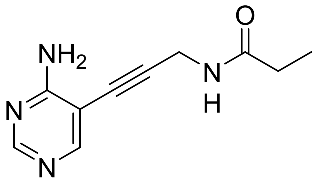

Compound **13** was synthesized according to the general method B. The crude product was purified by preparative HPLC (Condition A). **13** was obtained as a white solid, (24 mg, 0.12 mmol, 22%). ^{1}H NMR (500 MHz, DMSO-d_6) δ 8.77 (s, 1H), 8.28 (s, 1H), 4.20 (s, 2H), 2.13 (d, *J*=5.0, 2H), 1.01 (t, *J*=10.0, 5.0). ^{13}C NMR (126 MHz, DMSO-d_6) δ 174.28, 158.32, 157.69, 150.34, 121.23, 86.24, 79.06, 43.96, 29.86,

10.76. Purity via HPLC: 100%; MS (ESI+) *m/z* 204 [*M*+H]+; HRMS (ESI+) *m/z* calcd for $C_{10}H_{12}N_4O$ [*M*+H]+: 204.1011 found 204.0988.

N-(3-(4-amino-2-oxopyrimidin-1(2H)-yl)prop-2-yn-1-yl)cyclopropanecarboxamide (**14**)

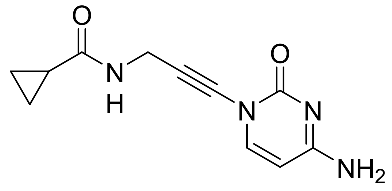

Compound **14** was synthesized according to the general method B. The crude product was purified by preparative HPLC (Condition B). **14** was obtained as a white solid, (38 mg, 0.16 mmol, 35%). ^{1}H NMR (500 MHz, DMSO-d_6) δ 8.52 (s, 1H), 7.70 (s, 1H), 4.12 (s, 2H), 1.57 (t, *J*=5.0, 10.0, 1H), 0.69 (d, *J*=5, 4H). ^{13}C NMR (126 MHz, DMSO-d_6) δ 172.35, 163.86, 151.88, 140.08, 97.43, 95.66, 85.22, 43.54, 15.12, 8.54. Purity via HPLC: 100%; MS (ESI+) *m/z* 232 [*M*+H]+; HRMS (ESI+) *m/z* calcd for $C_{11}H_{12}N_4O_2$ [*M*+H]+: 232.0960 found 232.0887.

N-(3-(4-amino-2-oxopyrimidin-1(2H)-yl)propyl)cyclopropanecarboxamide (**15**)

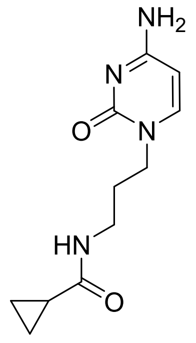

Compound **15** was synthesized according to the general method B. The crude product was purified by preparative HPLC (Condition A). **15** was obtained as a yellow oil, (31 mg, 0.13 mmol, 28%). ^{1}H NMR (500 MHz, DMSO-d_6) δ 8.08 (s, 1H), 7.15 (s, 1H), 3.79 (d, *J*=5.0, 2H), 3.07-3.07 (m, 2H), 1.99-1.79 (m, 1H), 1.77 (m, 1H), 1.50 (m, 1H), 0.85 (m, 2H), 0.65 (m, 2H). ^{13}C NMR (126 MHz, DMSO-d_6) δ 174.83, 165.24, 150.40, 145.62, 93.72, 47.34, 45.13, 25.62, 15.87, 9.64. Purity via HPLC: 100%; MS (ESI+) *m/z* 236 [*M*+H]+; HRMS (ESI+) *m/z* calcd for $C_{11}H_{16}N_4O_2$ [*M*+H]+: 236.1273 found 236.0998

N-(3-(4-amino-2-oxo-1,2-dihydropyrimidin-5-yl)propyl)cyclopropanesulfonamide (**16**)

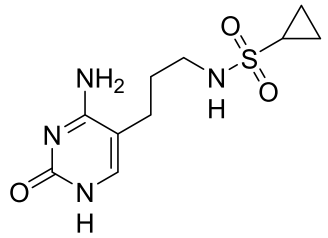

Compound **16** was synthesized according to the general method B. The crude product was purified by preparative HPLC (Condition A). **16** was obtained as a yellow oil, (64 mg, 0.24 mmol, 58%).^{1}H NMR (500 MHz, DMSO-d_6) δ 7.24 (s, 1H), 3.02 (m, 2H), 2.58 (m, 2H), 2.34 (m, 2H), 1.64 (t, *J*=10.0, 5.0, 1H), 0.98 (m, 4 H). ^{13}C NMR (126 MHz, DMSO-d_6) δ 165.39, 151.23, 139.28, 136.83, 48.96, 42.78, 29.67, 25.34, 8.62. Purity via HPLC: 100%; MS (ESI+) *m/z* 272 [*M*+H]+; HRMS (ESI+) *m/z* calcd for $C_{10}H_{16}N_4O_3S$ [*M*+H]+: 272.0943 found 272.0891.

N-(3-(4-amino-2-oxo-1-(tetrahydrothiophen-2-yl)-1,2-dihydropyrimidin-5-yl)prop-2-yn-1-yl)cyclopropanecarboxamide **(17)**

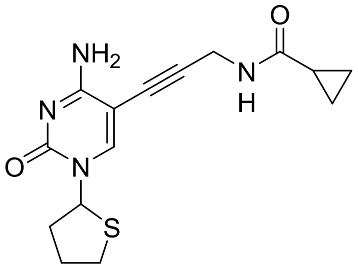

Compound **17** was synthesized according to the general method B. The crude product was purified by preparative HPLC (Condition A). 1**7** was obtained as a white solid. (20 mg, 0.06 mmol, 10%). ^{1}H NMR (500 MHz, DMSO-d$_6$) δ 8.18 (s, 1H), 6.22 (dd, *J*= 5.6, 5.8 Hz, 1H), 4.18 (d, *J*= 5.0 Hz, 2H), 3.32 (ddd, *J*= 6.4, 6.0, 4.1 Hz, 1H), 2.91 (ddd, *J*= 6.4, 6.4, 3.7 Hz, 1H), 2.28-2.23 (m, 1H), 2.13-2.01 (m, 3H), 1.63 (tt, *J*= 5.3, 5.4 Hz, 1H), 0.75-0.73 (m, 4 H). ^{13}C NMR (126 MHz, DMSO-d$_6$) δ 173.48, 155.12, 151.55, 142,76, 120.68, 85.12, 80.76, 63.02, 42.88, 31.53, 30.86, 30.11, 15.68, 7.66. Purity via HPLC: 98%; MS (ESI+) *m/z* 319 [*M*+H]+; HRMS (ESI+) *m/z* calcd for $C_{15}H_{18}N_4O_2S$ [*M*+H]$_+$: 319.1150 found 319.1218.

3.5 Spectra:

N-(3-(4-amino-2-oxo-1,2-dihydropyrimidin-5-yl)prop-2-yn-1-yl)cyclopropanecarboxamide (**3**)

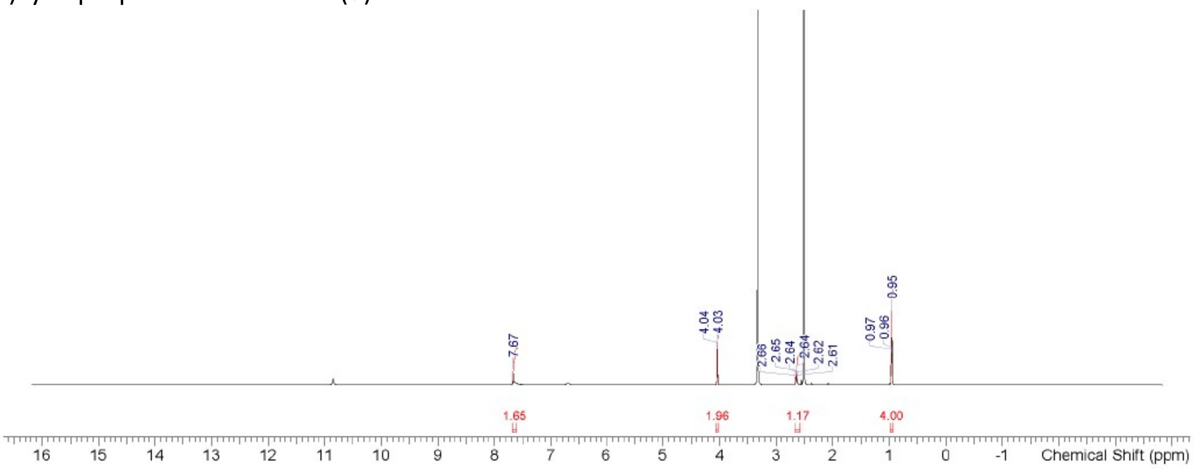

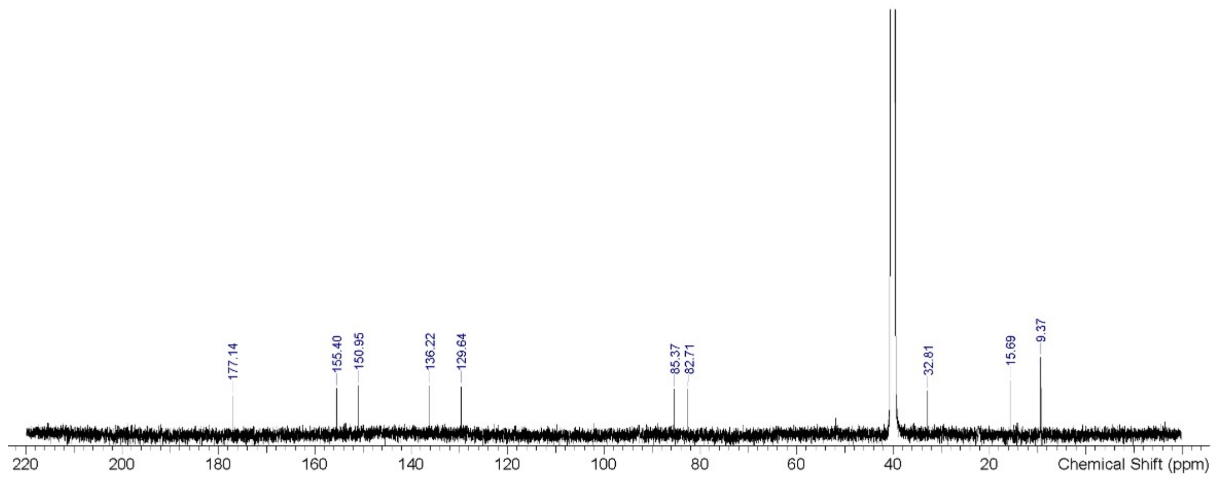

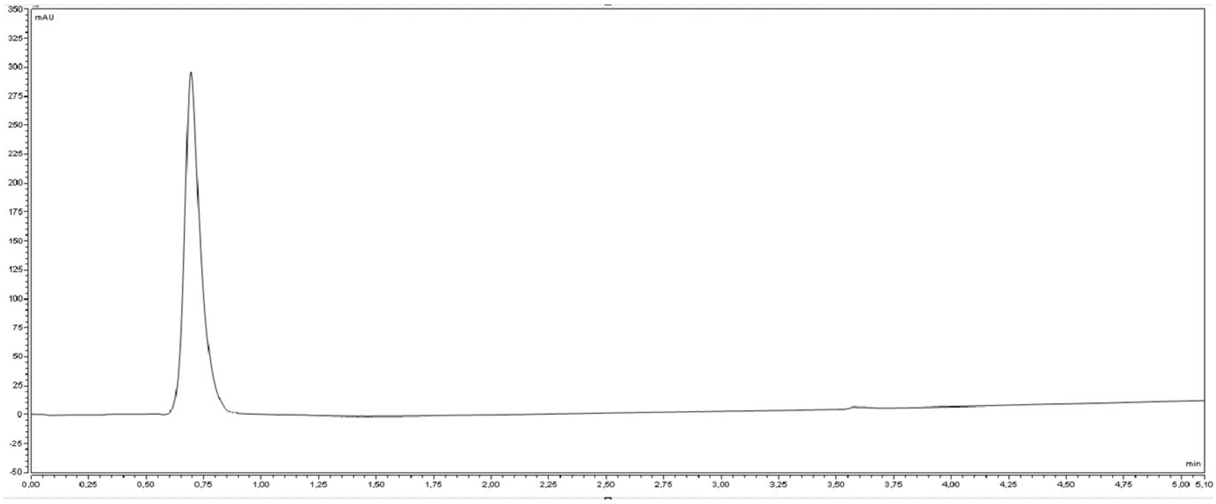

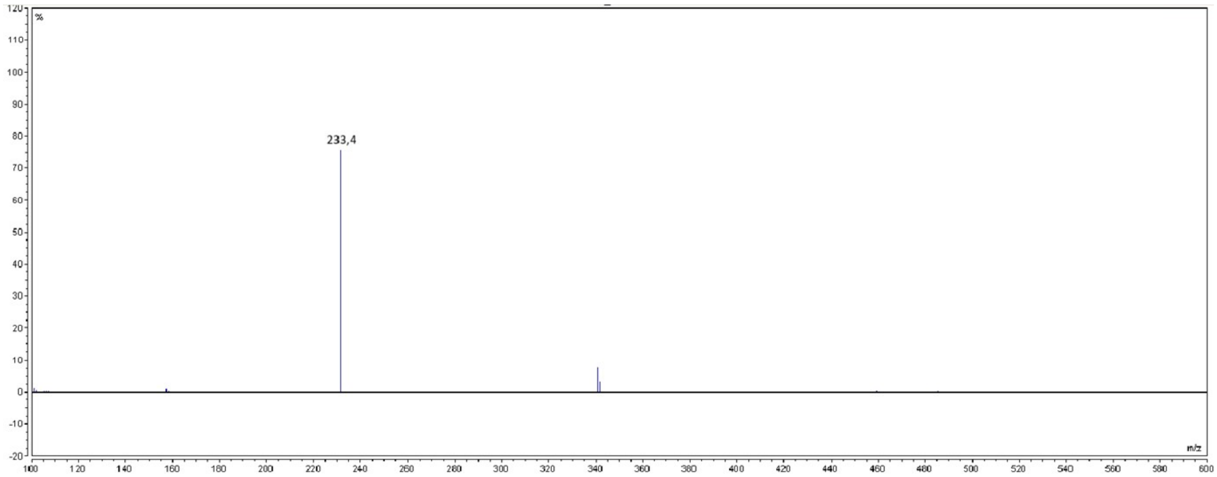

N-(3-(4-amino-2-oxo-1,2-dihydropyrimidin-5-yl)prop-2-yn-1-yl)acetamide (177-8060) (**4**)

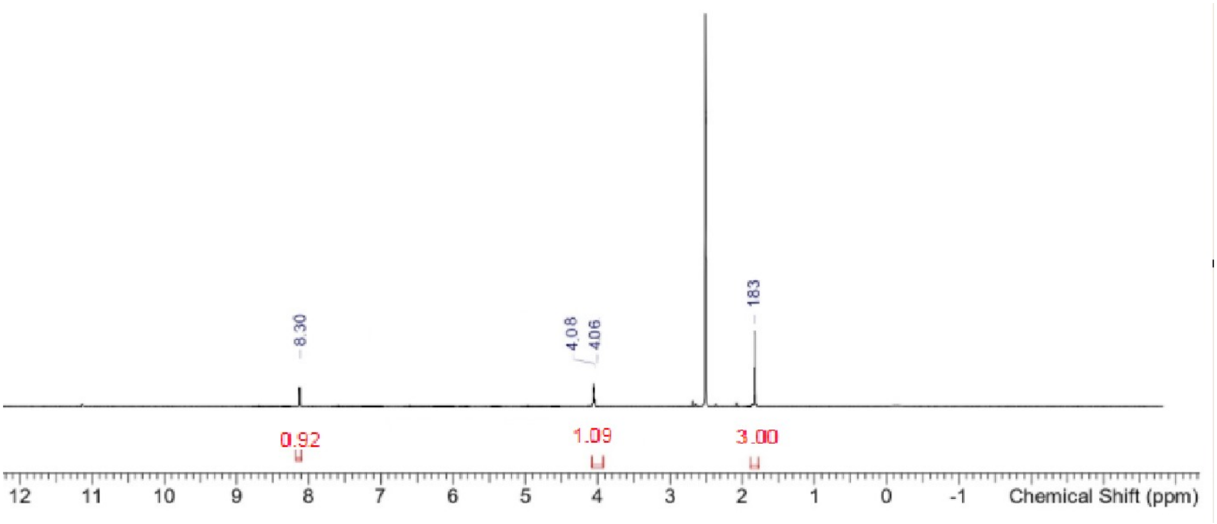
8.30
4.08
4.06
183
0.92
1.09
3.00
12 11 10 9 8 7 6 5 4 3 2 1 0 -1
Chemical Shift (ppm)

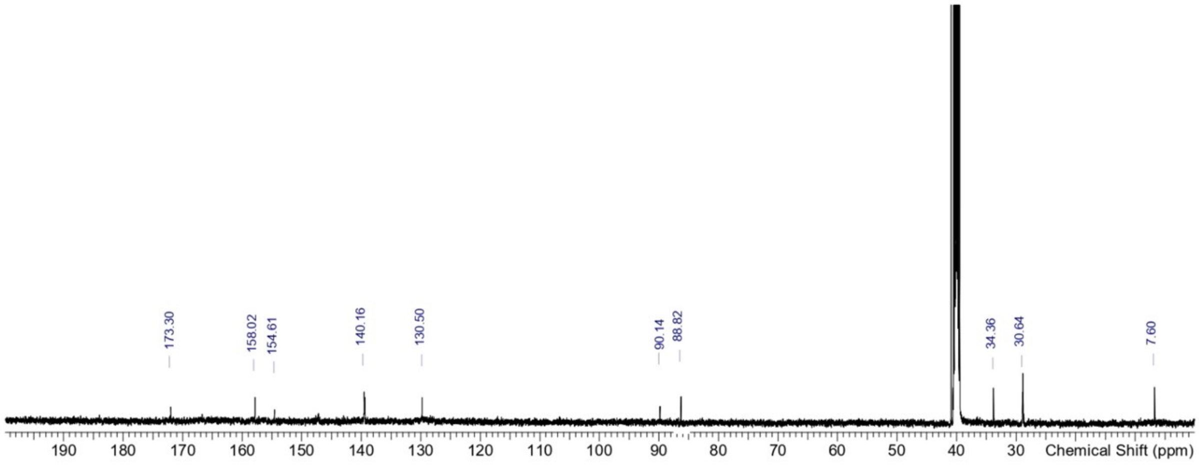
173.30
158.02
154.61
140.16
130.50
90.14
88.82
34.36
30.64
7.60
190 180 170 160 150 140 130 120 110 100 90 80 70 60 50 40 30
Chemical Shift (ppm)

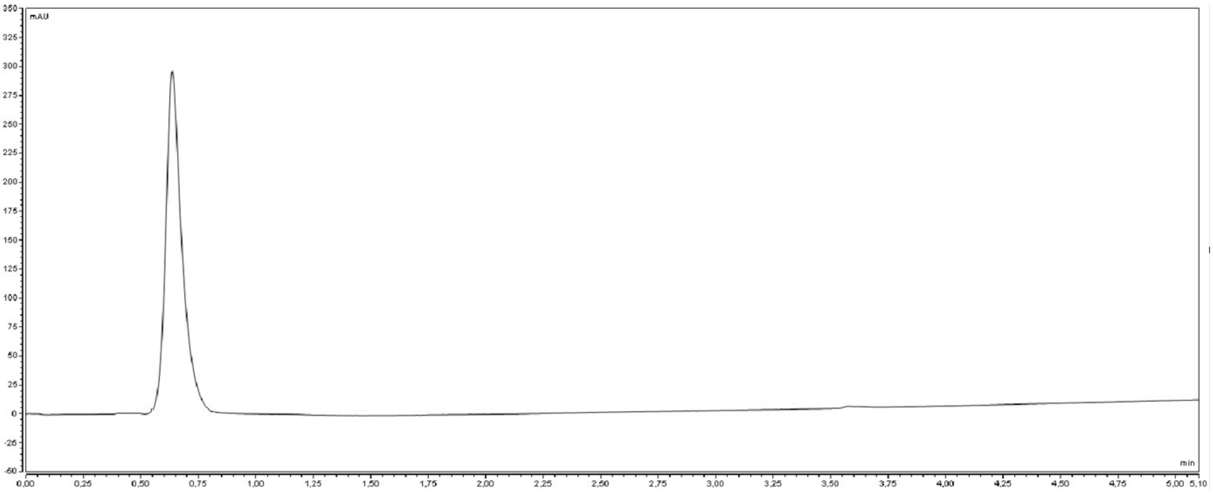
mAU
min

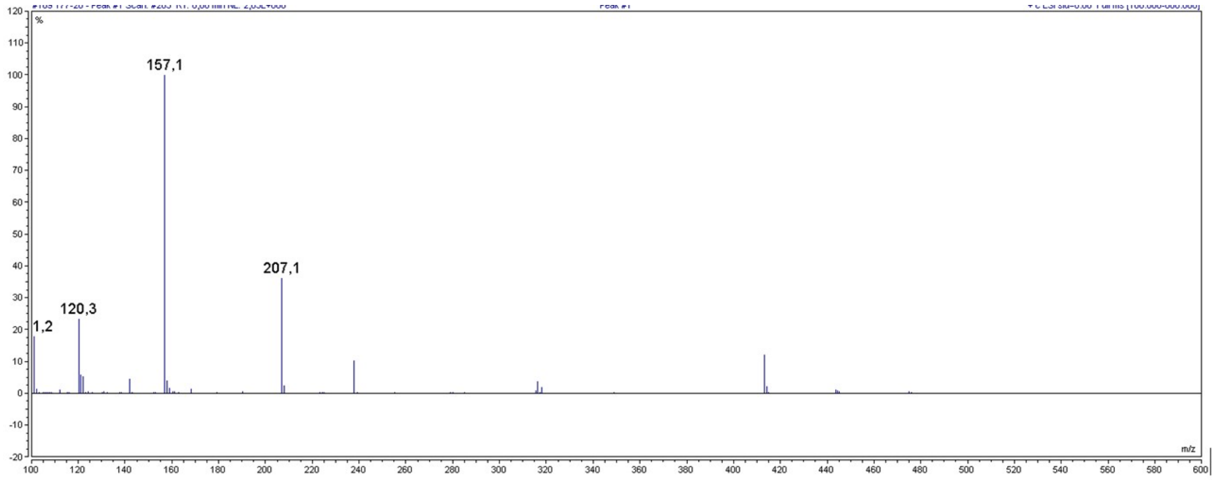

N-(3-(4-amino-2-oxo-1,2-dihydropyrimidin-5-yl)prop-2-yn-1-yl)propionamide (**5**)

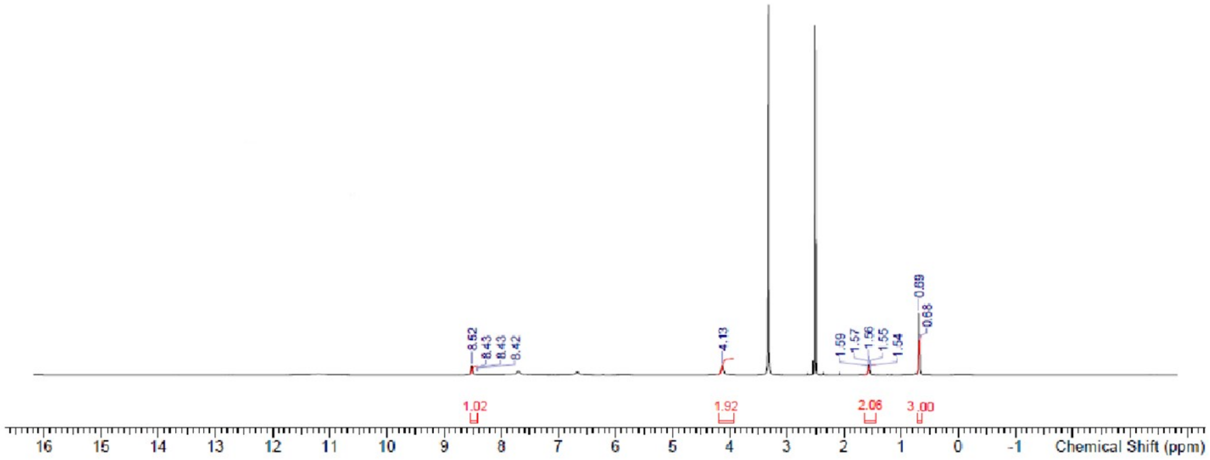

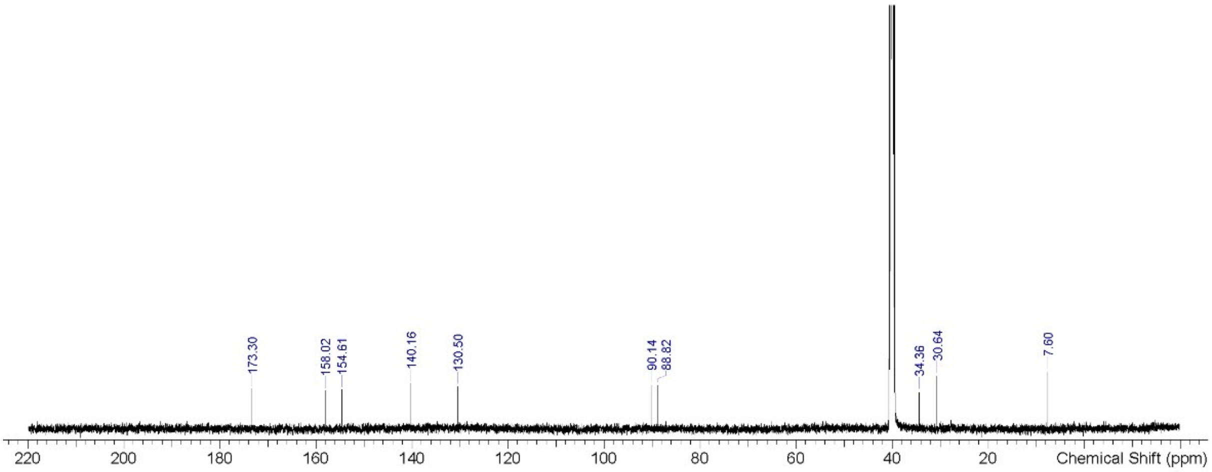

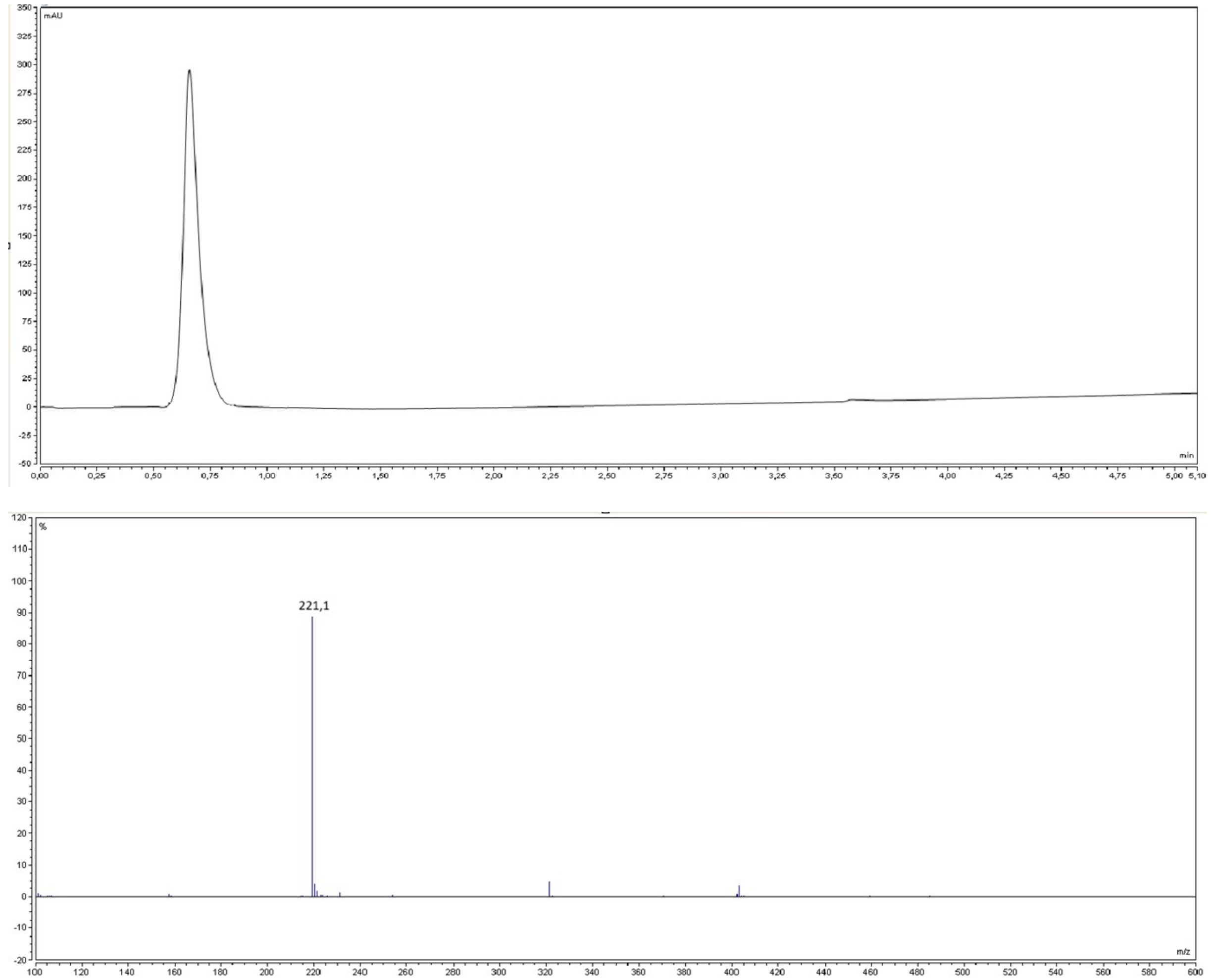

N-(3-(4-amino-2-oxo-1,2-dihydropyrimidin-5-yl)prop-2-yn-1-yl)pentanamide (**6**)

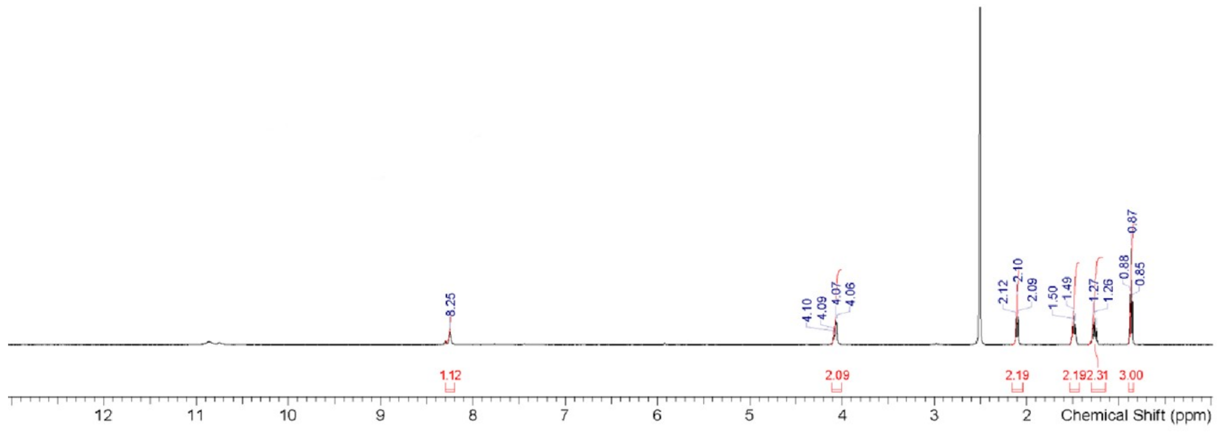

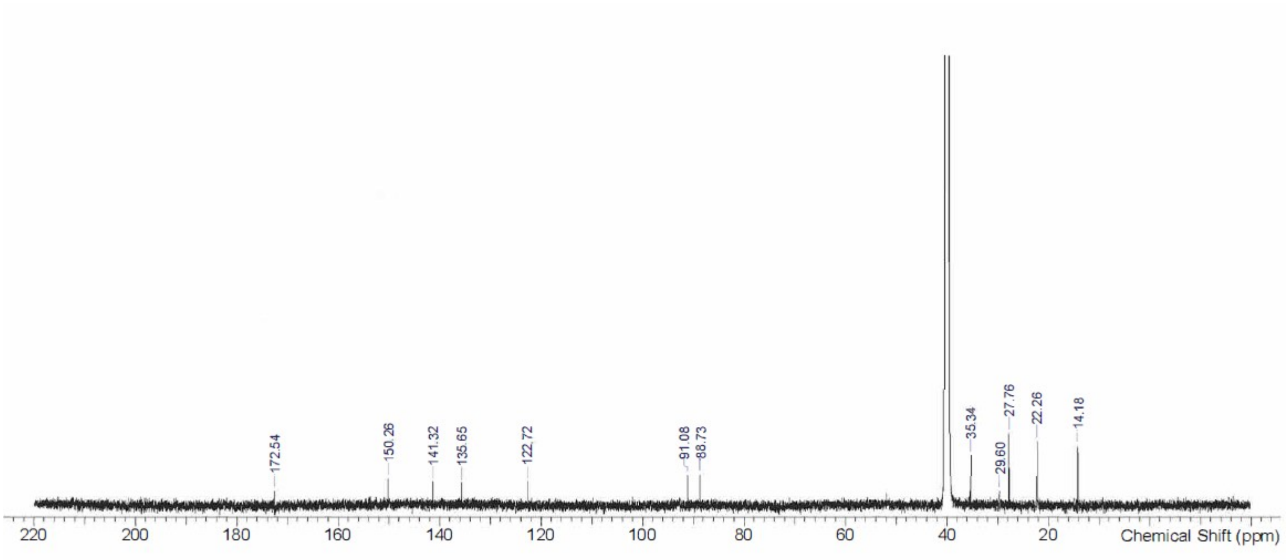
172.54
150.26
141.32
135.65
122.72
91.08
88.73
35.34
29.60
27.76
22.26
14.18
220 200 180 160 140 120 100 80 60 40 20
Chemical Shift (ppm)

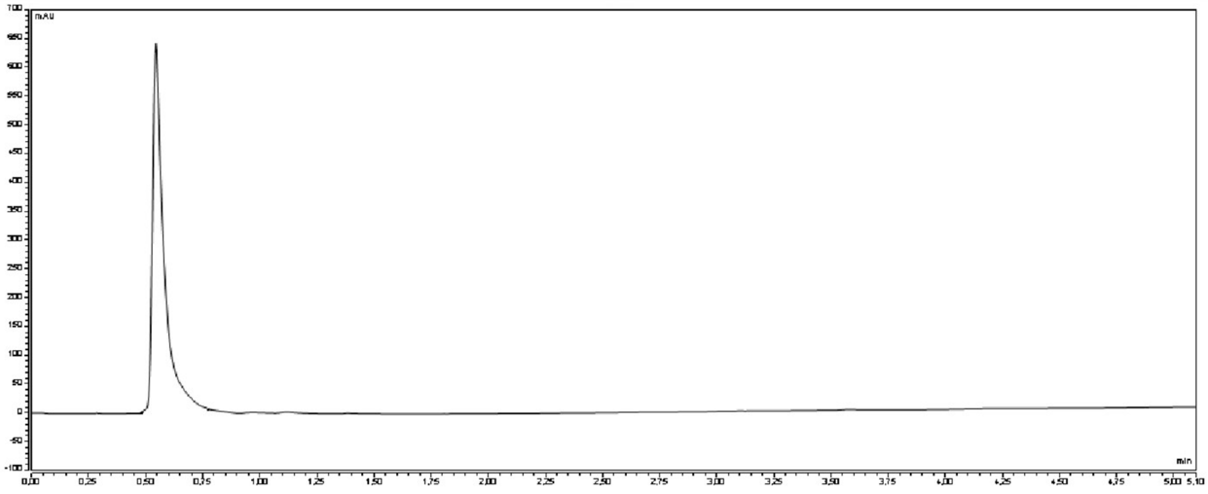
mAU
min

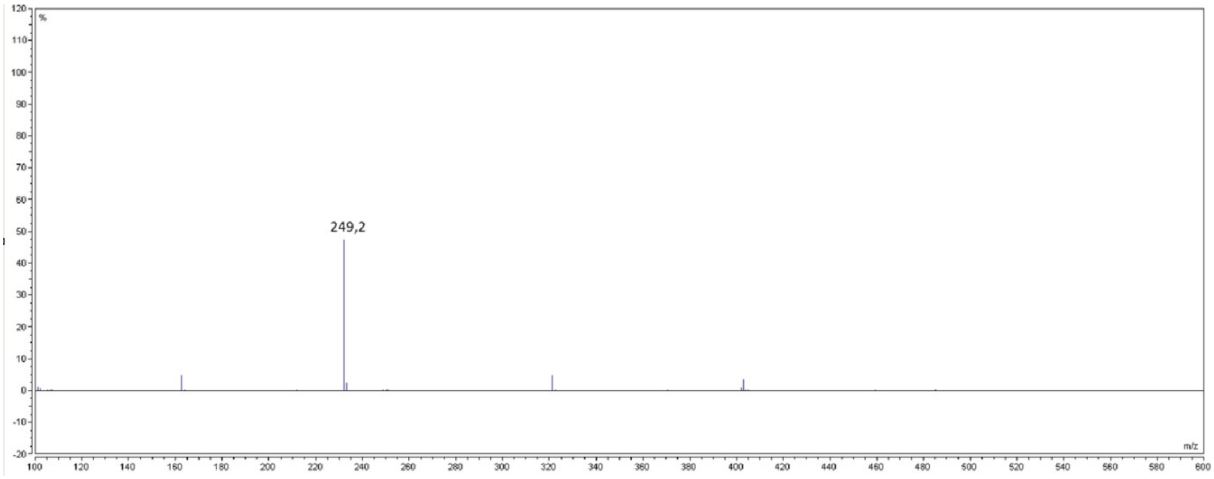
%
249,2
m/z

N-(3-(4-amino-2-oxo-1,2-dihydropyrimidin-5-yl)prop-2-yn-1-yl)pivalamide (**7**)

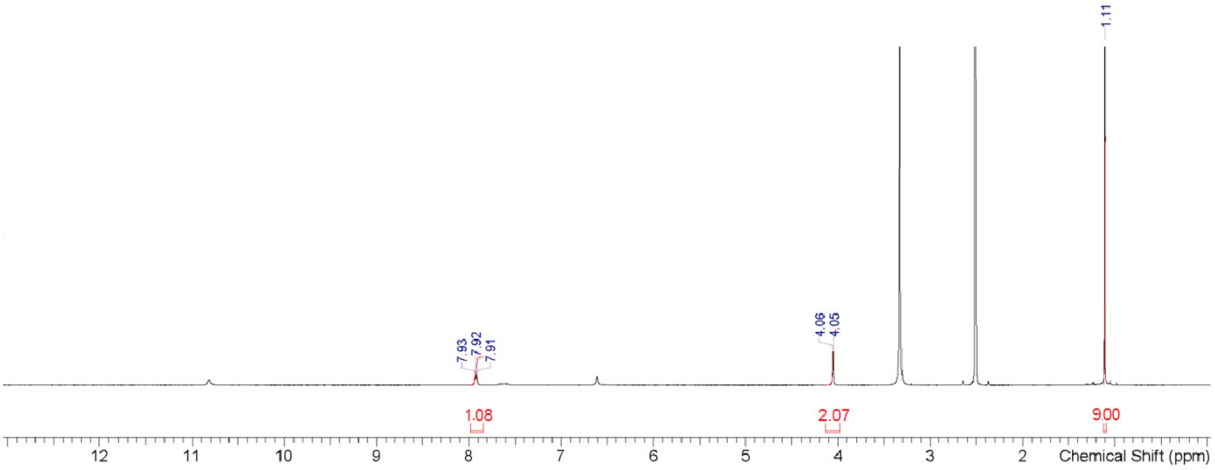

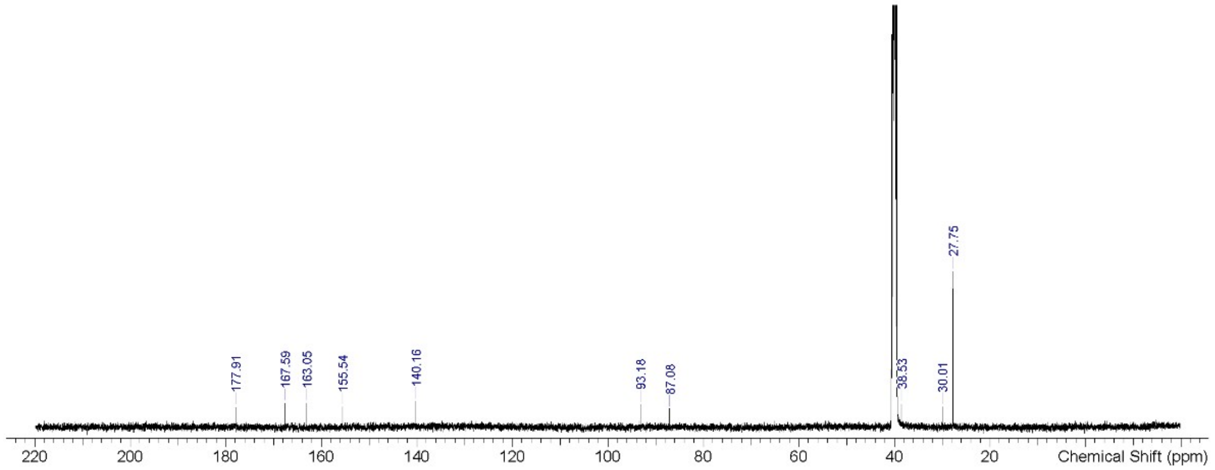

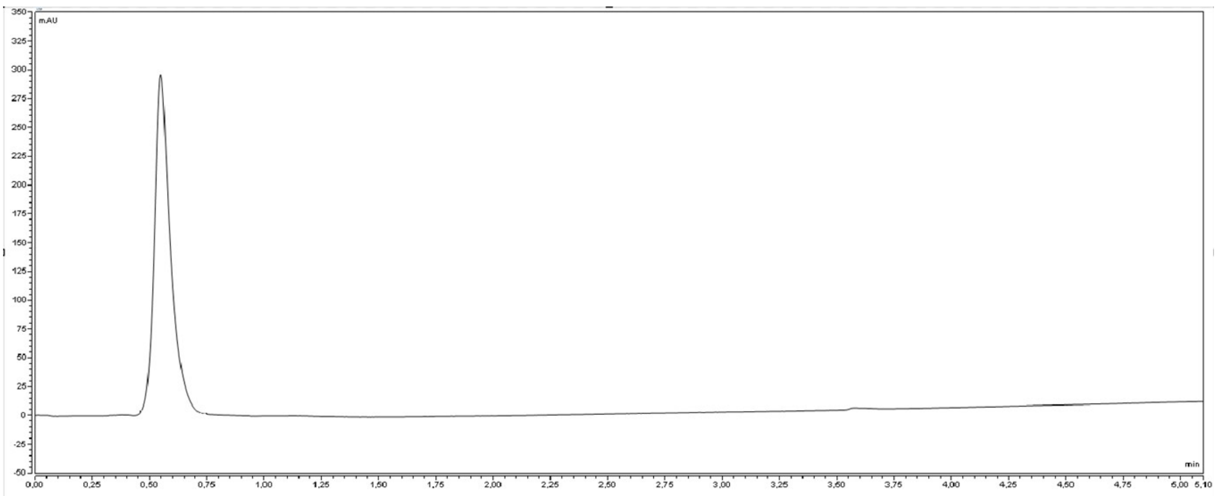

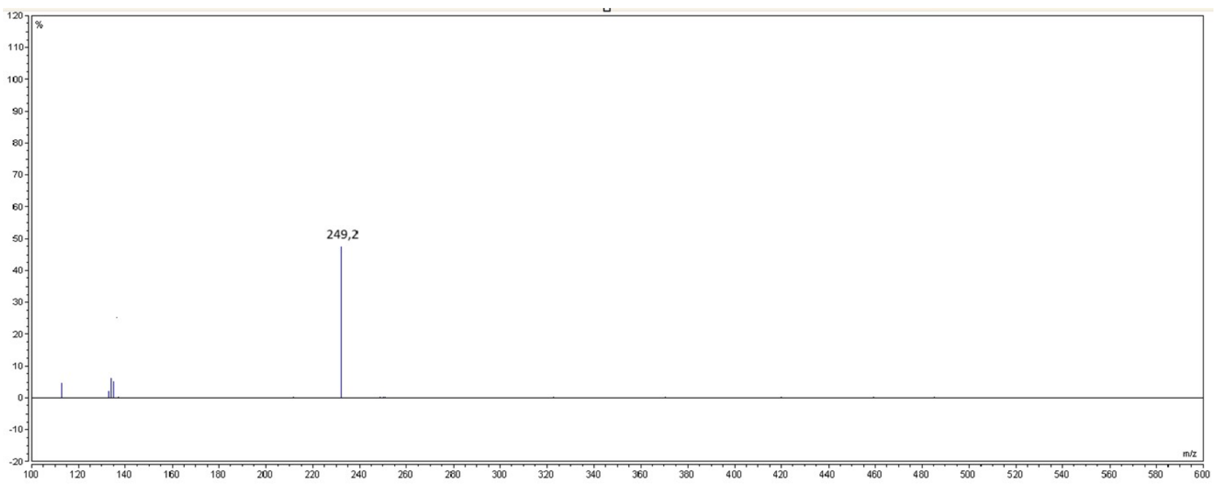

N-(3-(4-amino-2-oxo-1,2-dihydropyrimidin-5-yl)prop-2-yn-1-yl)cyclohexanecarboxamide **(8)**

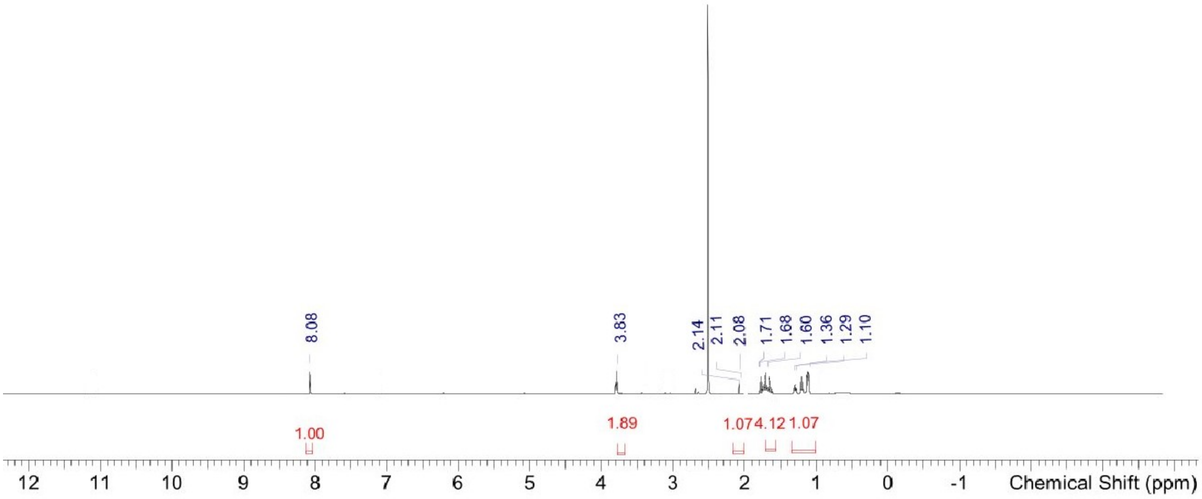

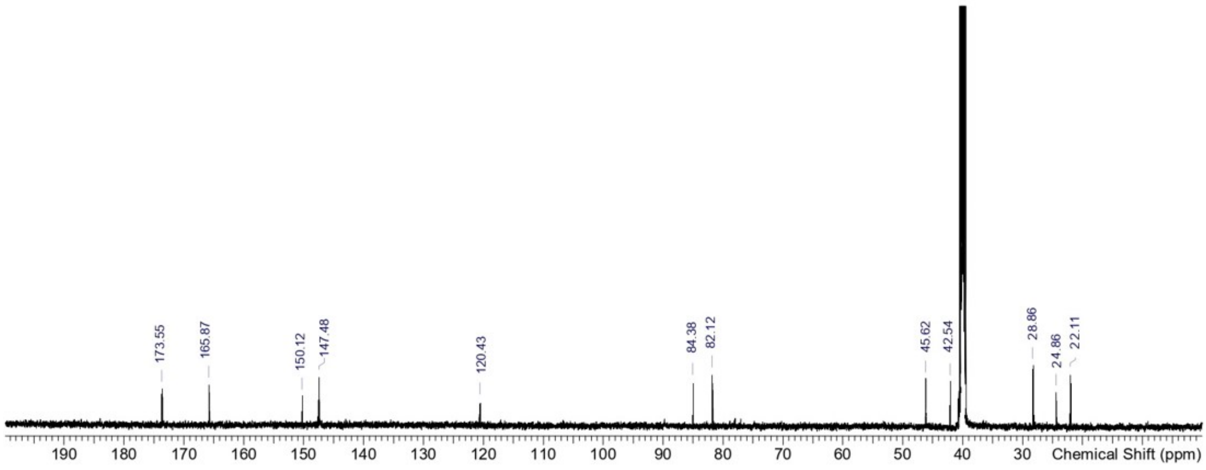

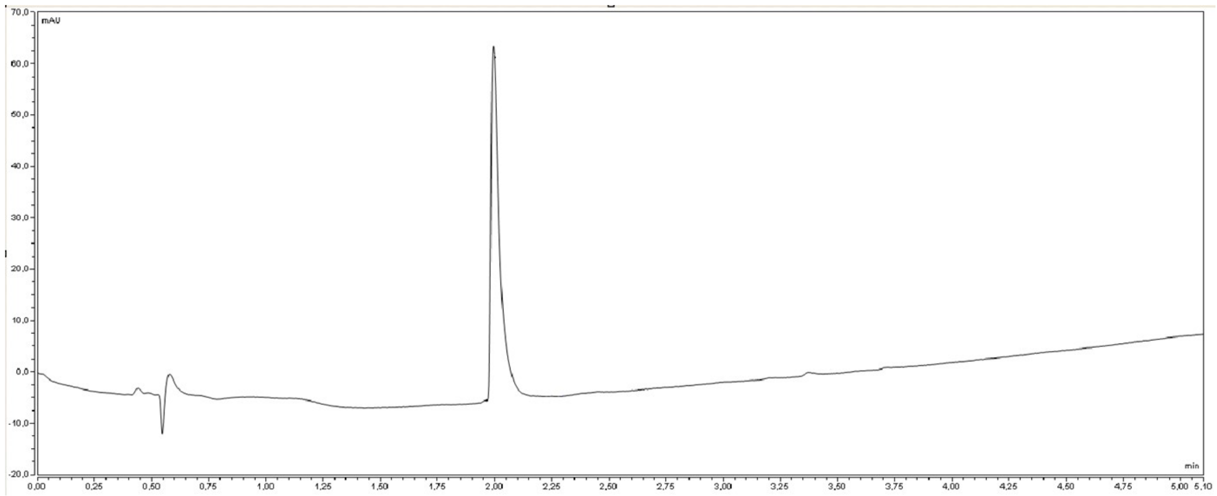

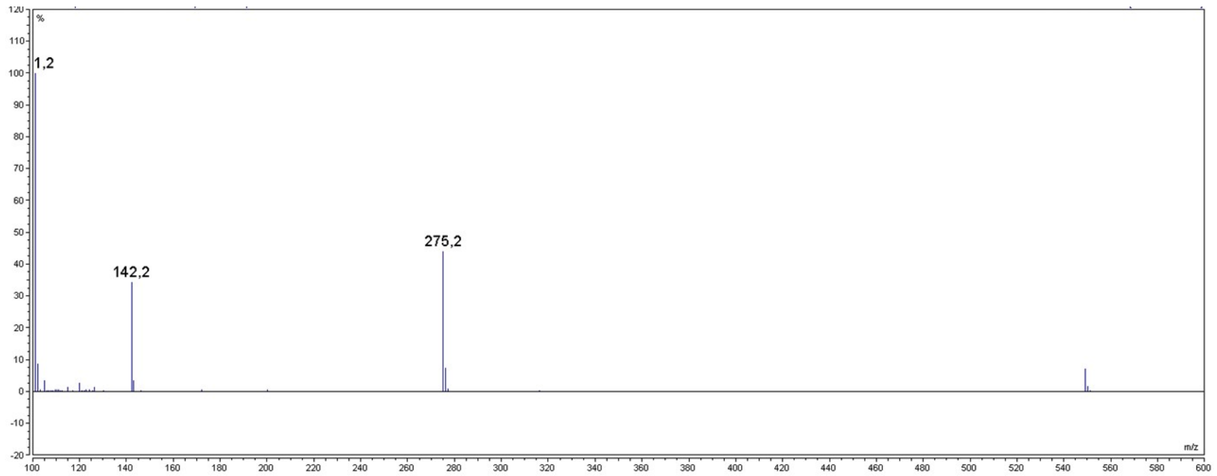

N-(3-(4-amino-2-oxo-1,2-dihydropyrimidin-5-yl)prop-2-yn-1-yl)acrylamide (**9**)

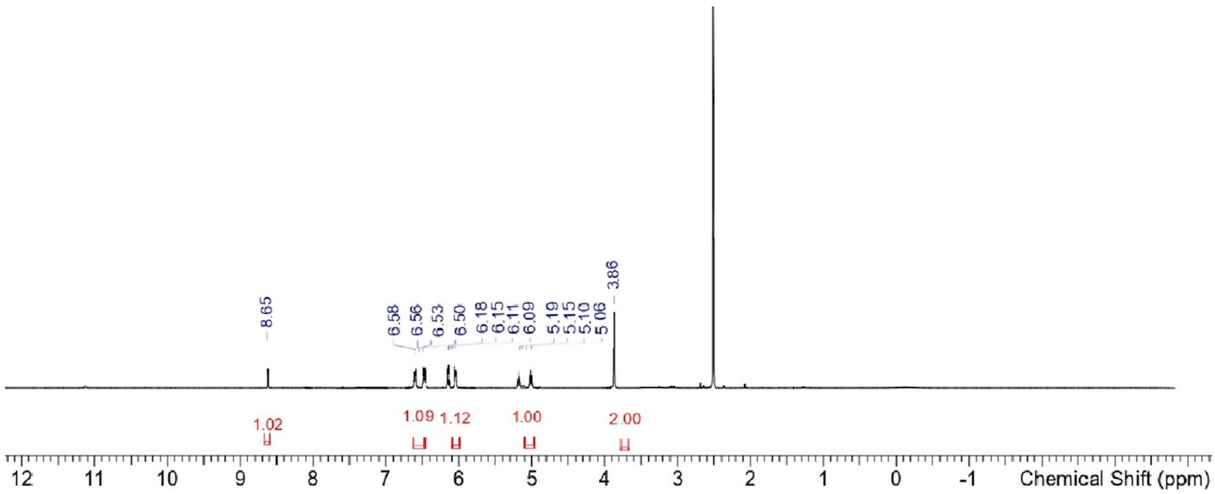

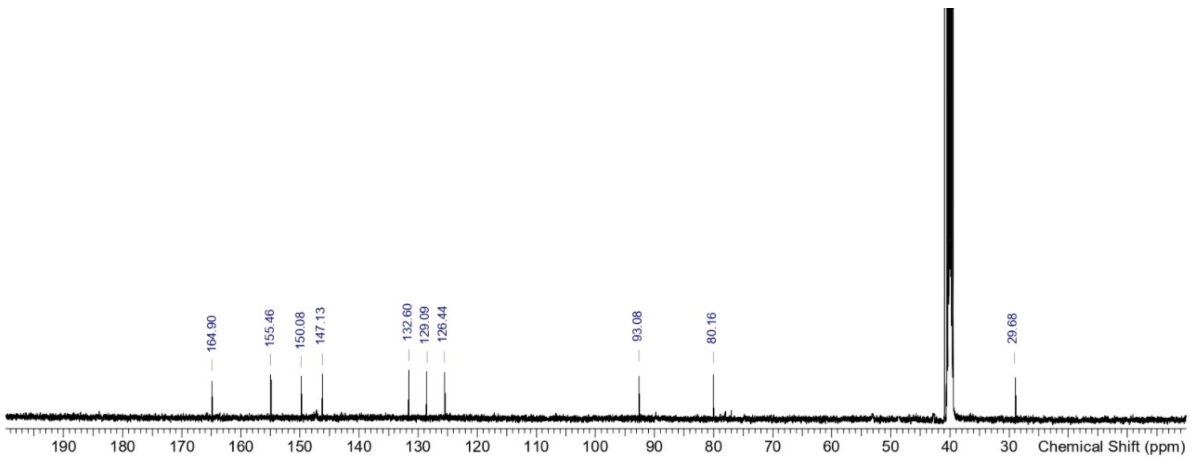

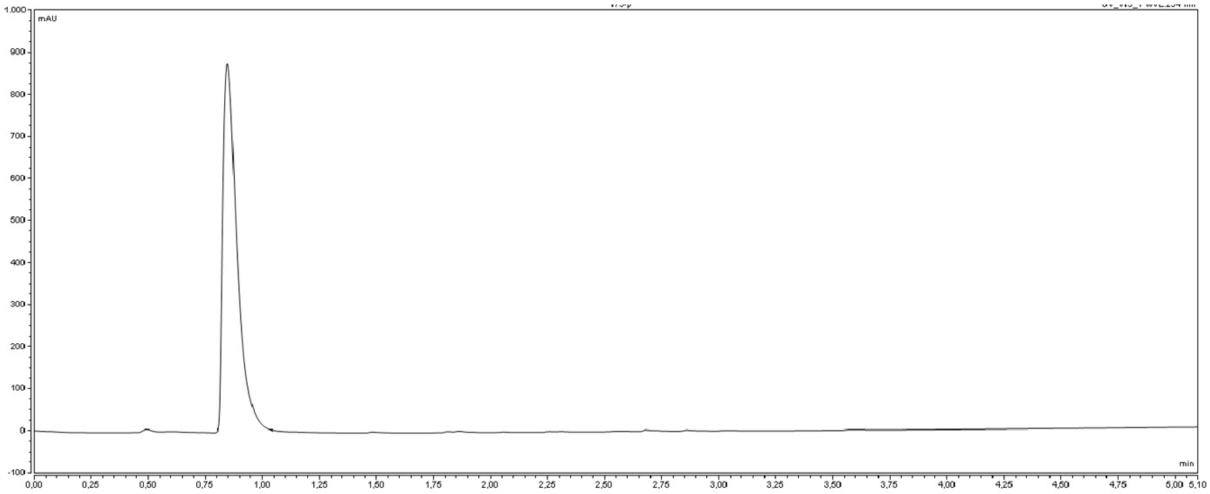

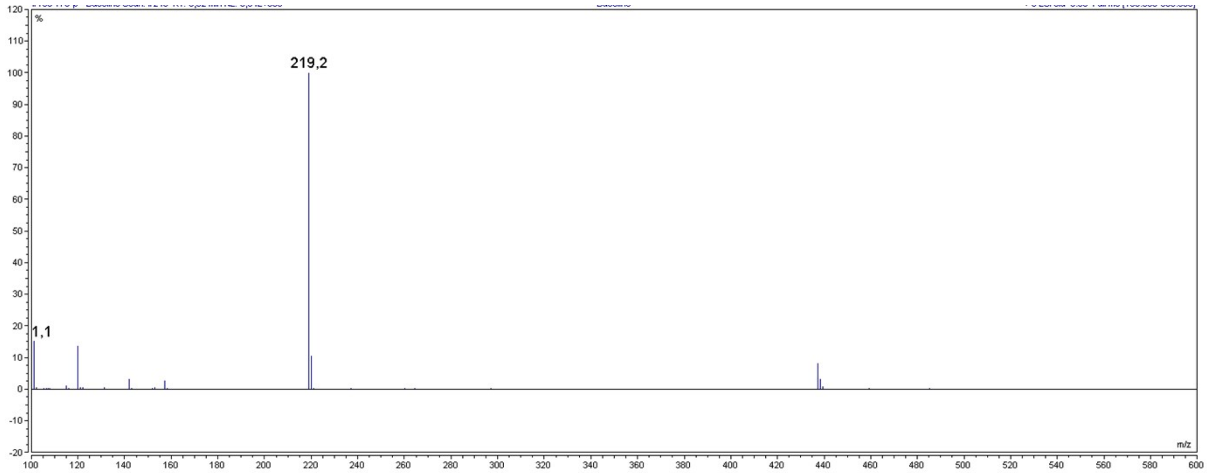

N-(3-(4-amino-2-oxo-1,2-dihydropyrimidin-5-yl)prop-2-yn-1-yl)-3,3,3-Trifluoropropanamide (**10**)

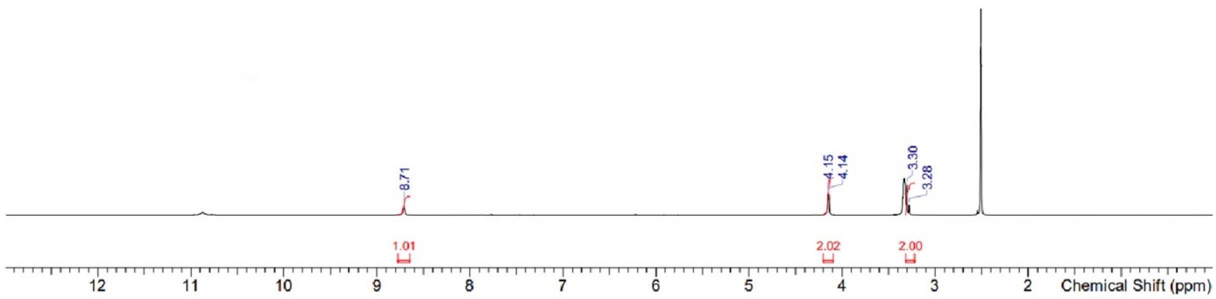
8.71
4.15
4.14
3.30
3.28
1.01
2.02
2.00
Chemical Shift (ppm)

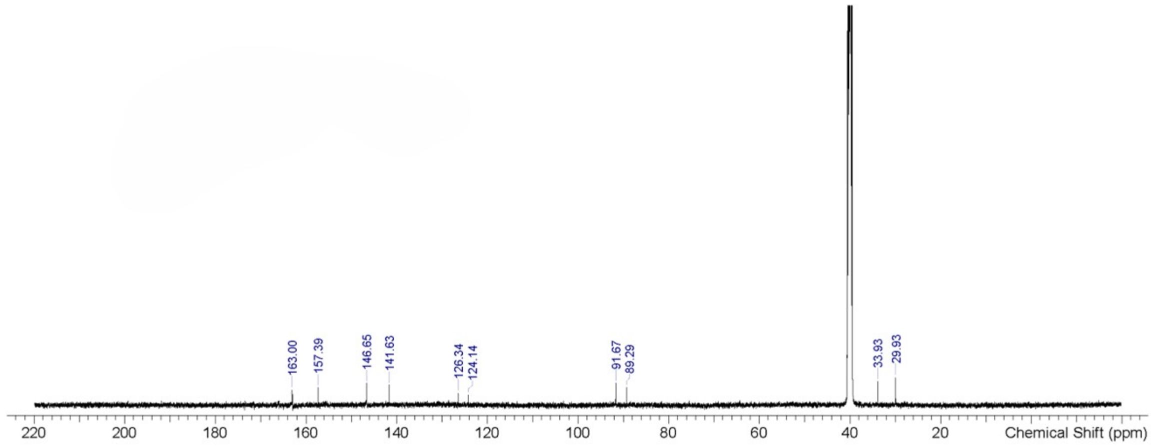
163.00
157.39
146.65
141.63
126.34
124.14
91.67
89.29
33.93
29.93
Chemical Shift (ppm)

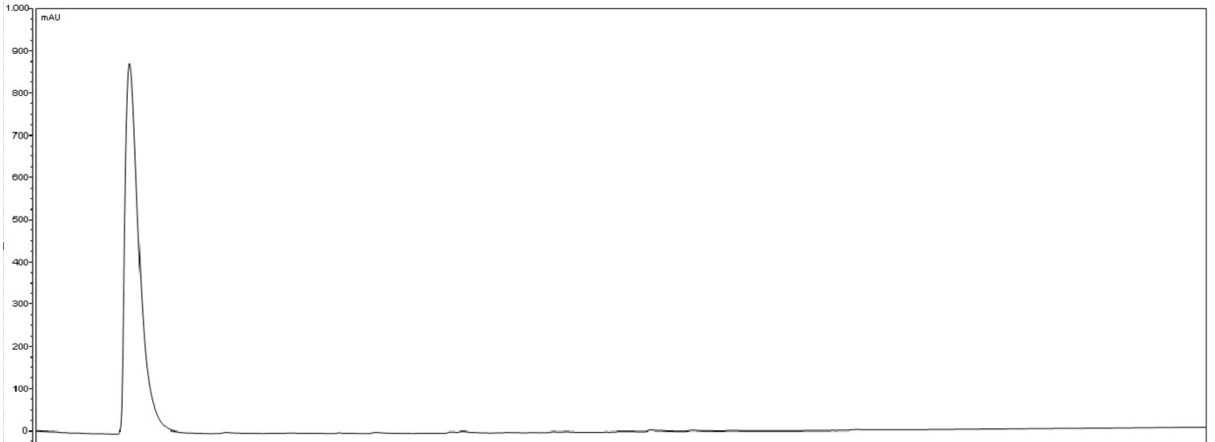
mAU
min

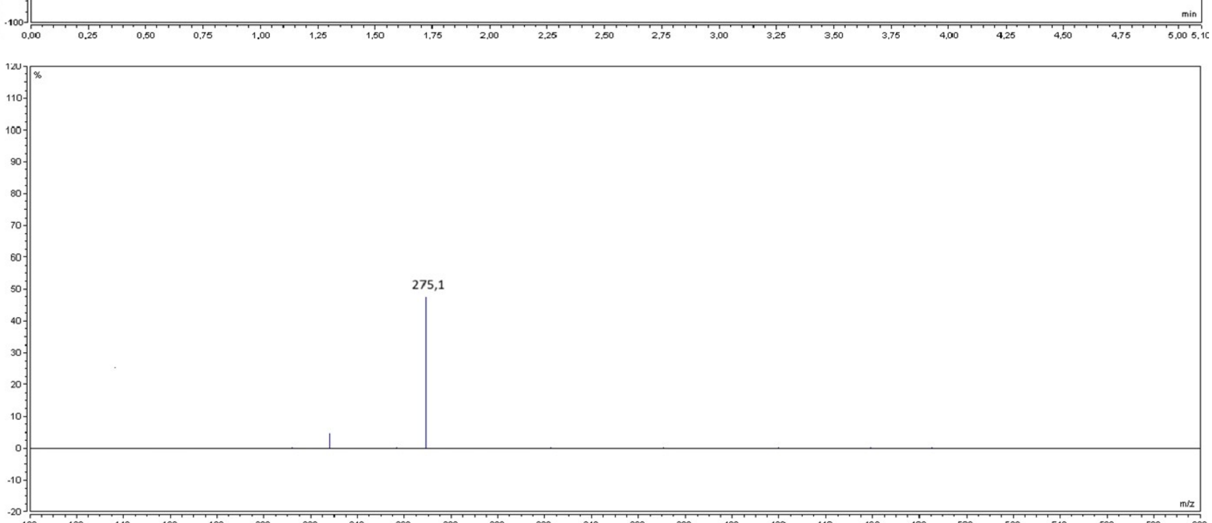
%
275,1
m/z

4-amino-5-(3-(piperazin-1-yl)prop-1-yn-1-yl)pyrimidin-2(1H)-one (**11**)

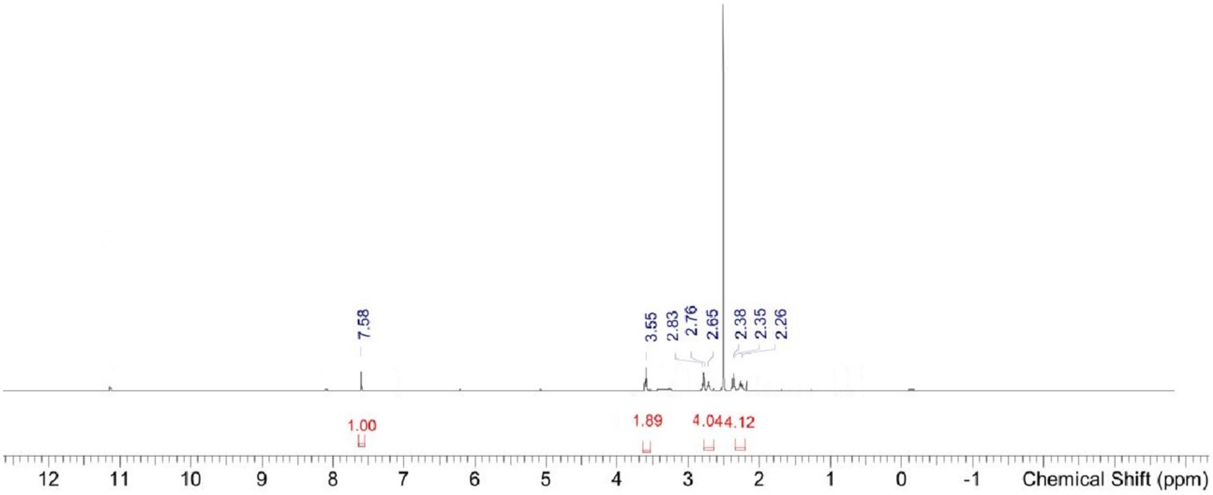

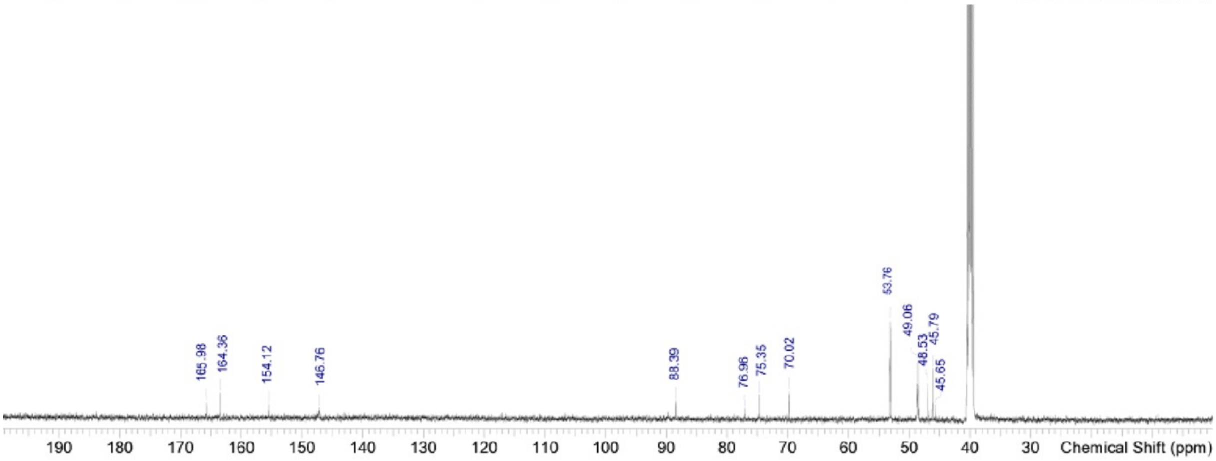

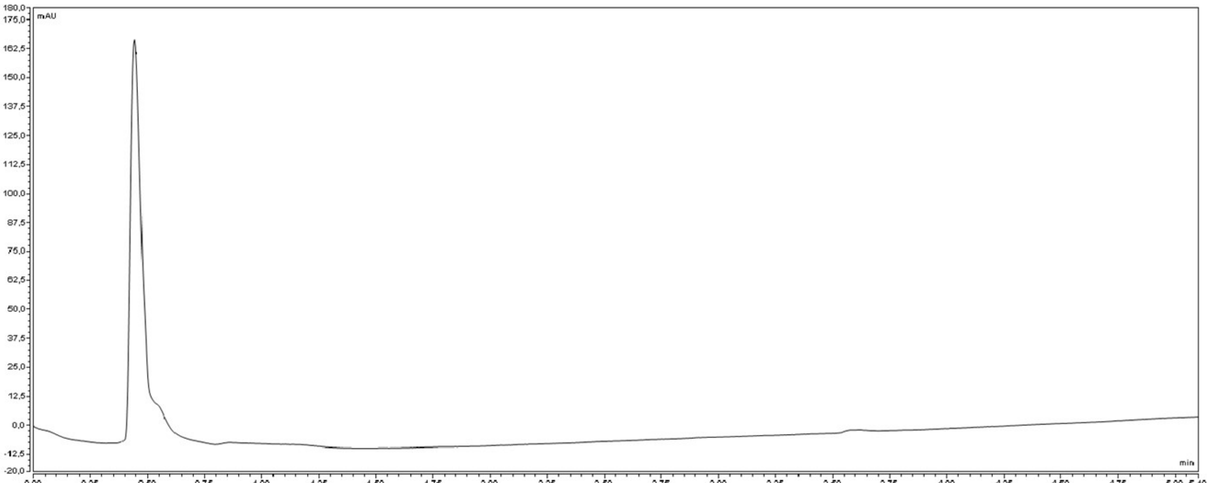

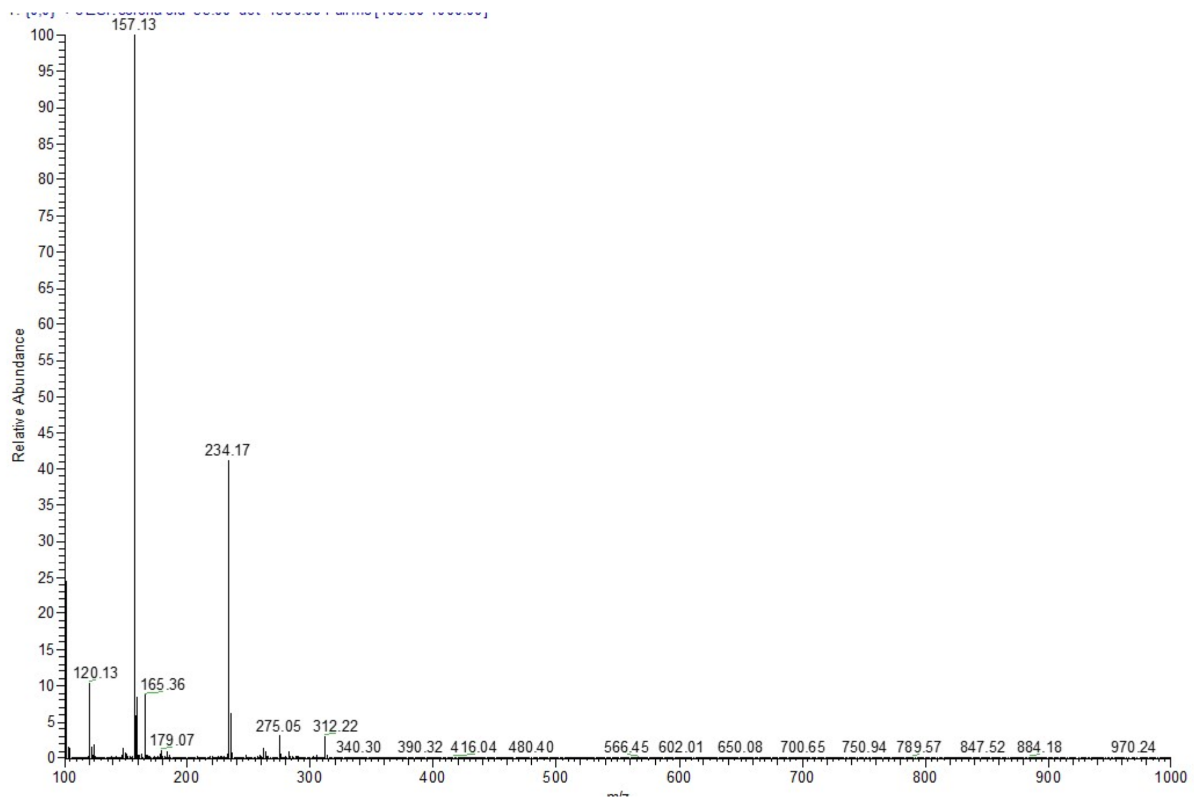

4-amino-5-(3-(4-methylpiperazin-1-yl)prop-1-yn-1-yl)pyrimidin-2(1H)-one **(12)**

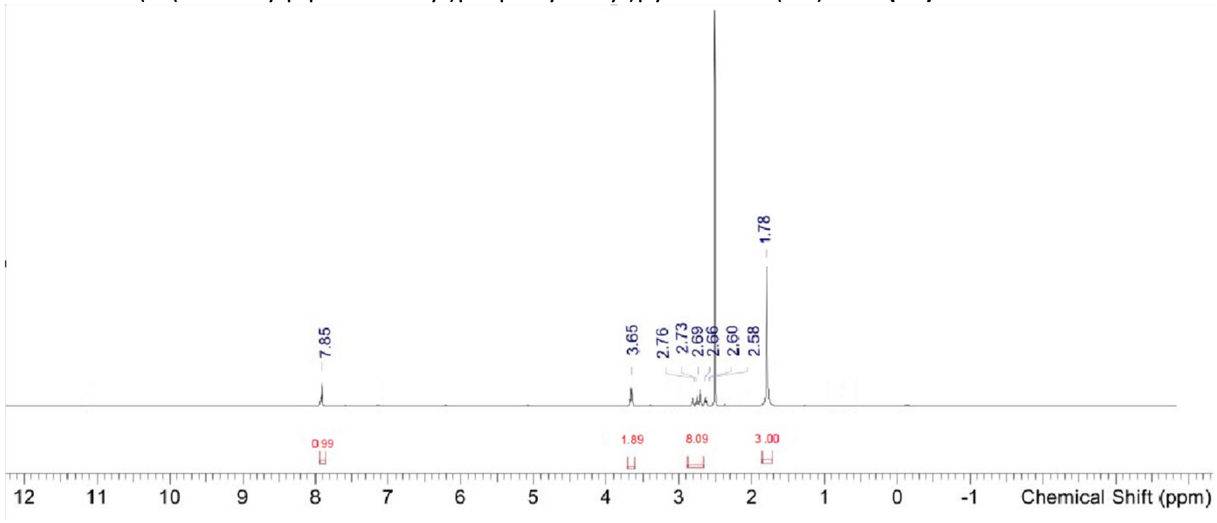

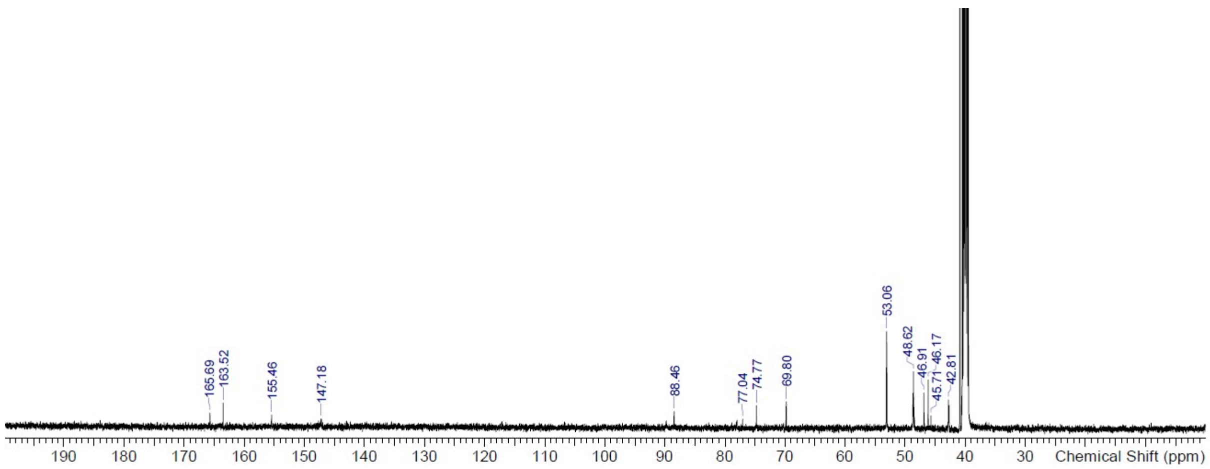

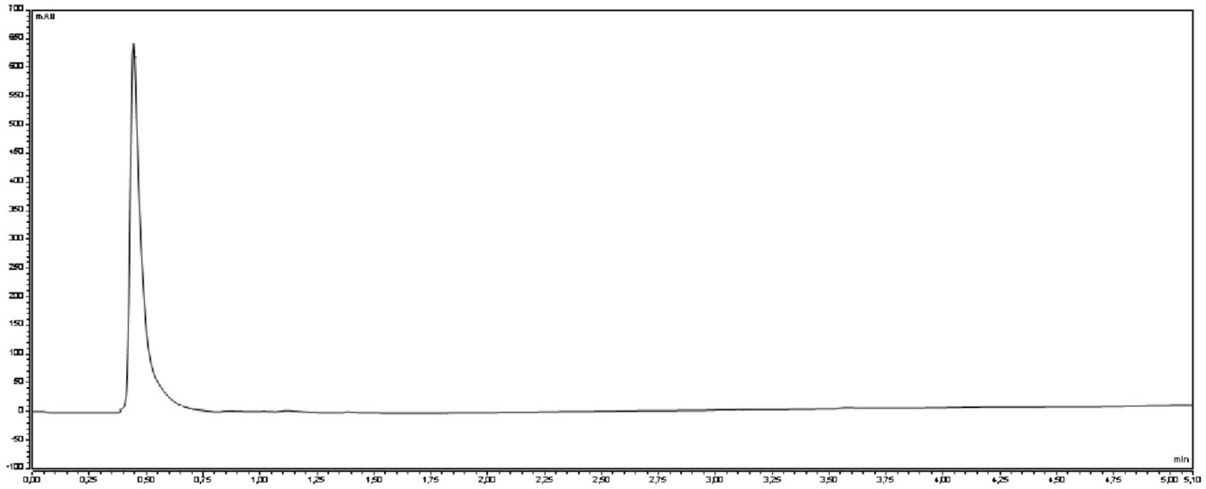

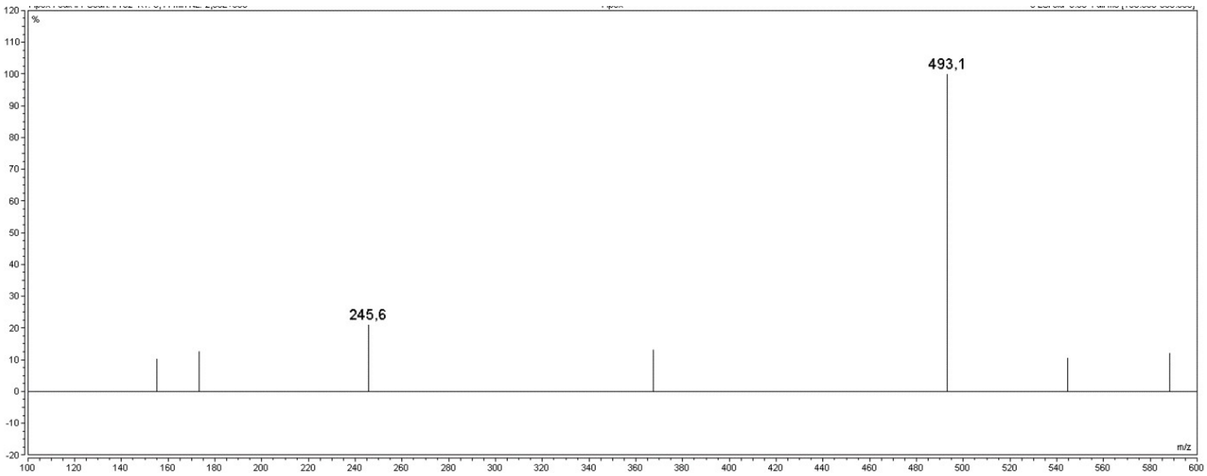

N-(3-(4-aminopyrimidin-5-yl)prop-2-yn-1-yl)propionamide **(13)**

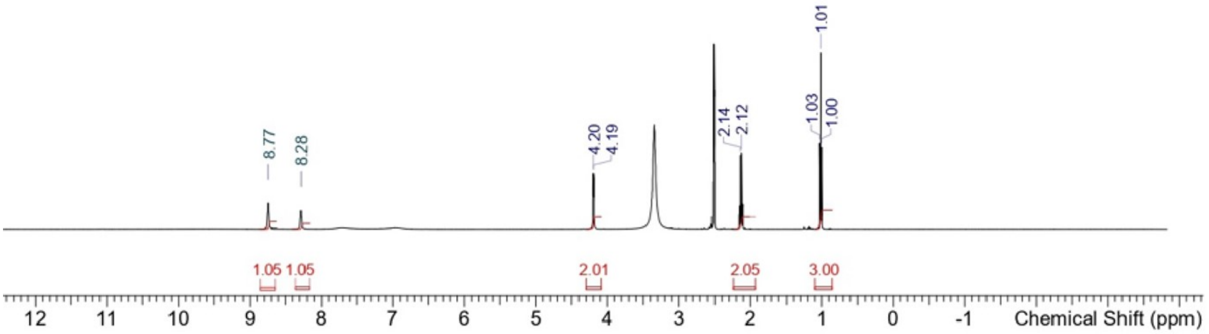
8.77
8.28
4.20
4.19
2.14
2.12
1.03
1.01
1.00
1.05
1.05
2.01
2.05
3.00
12 11 10 9 8 7 6 5 4 3 2 1 0 -1
Chemical Shift (ppm)

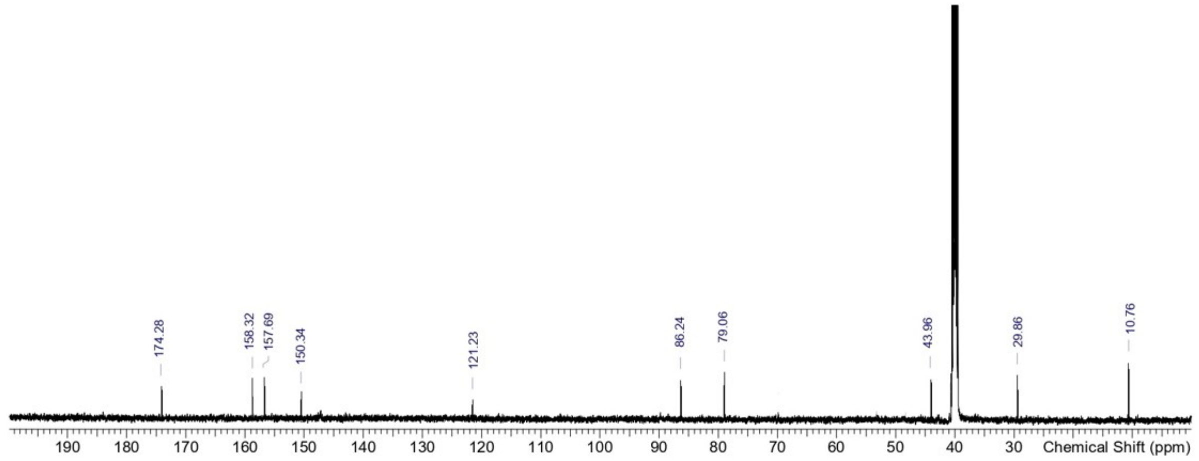
174.28
158.32
157.69
150.34
121.23
86.24
79.06
43.96
29.86
10.76
190 180 170 160 150 140 130 120 110 100 90 80 70 60 50 40 30
Chemical Shift (ppm)

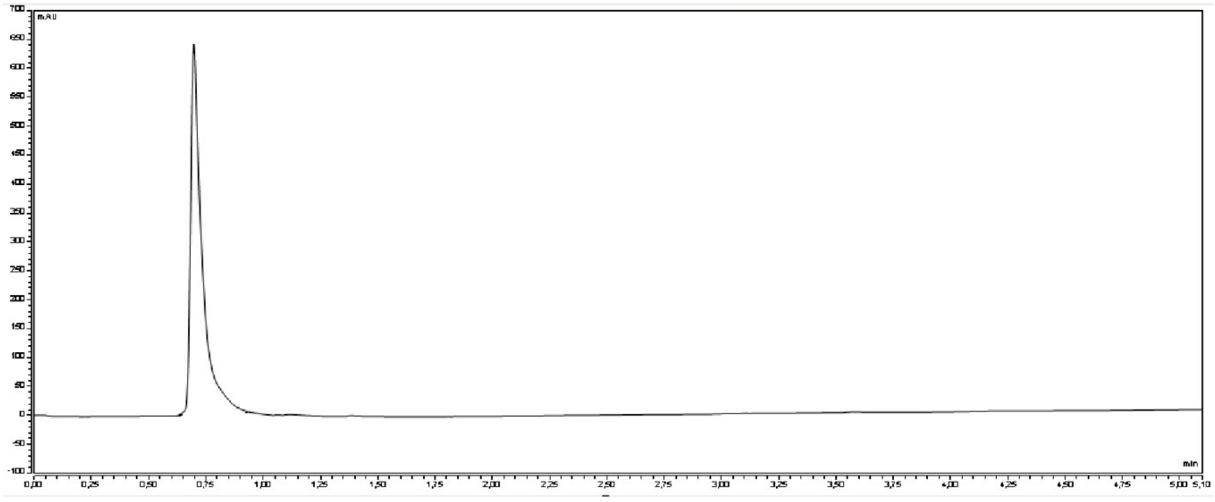
mAU
min

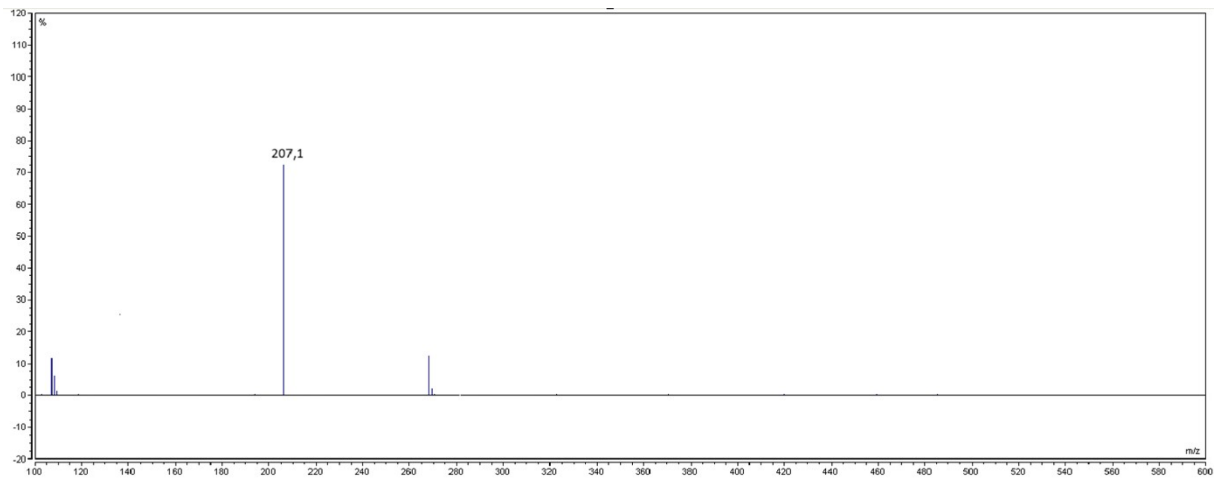

N-(3-(4-amino-2-oxopyrimidin-1(2H)-yl)prop-2-yn-1-yl)cyclopropanecarboxamide (**14**)

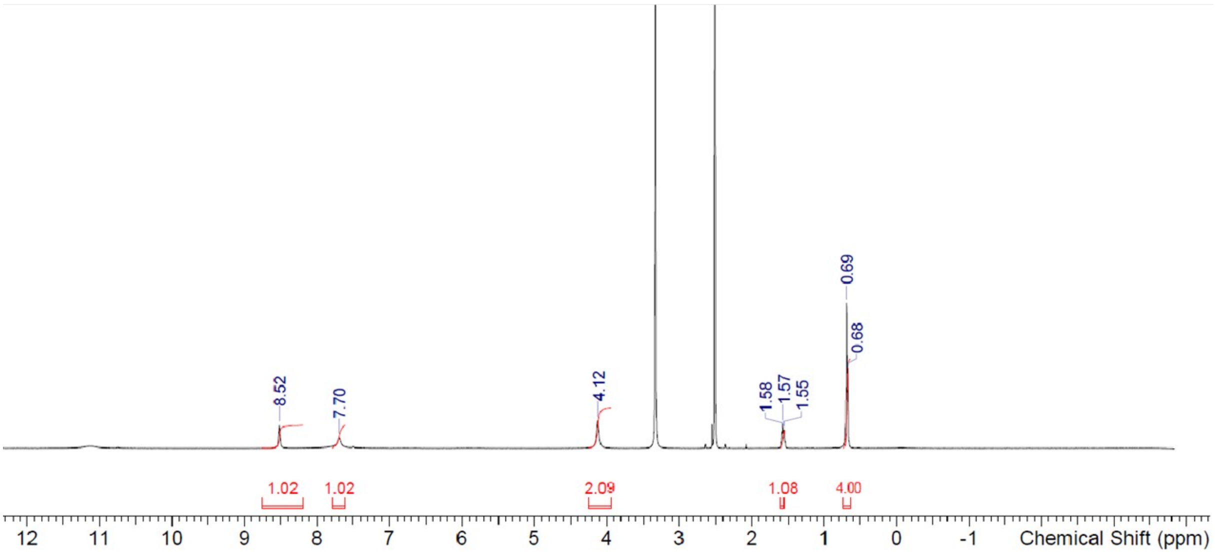

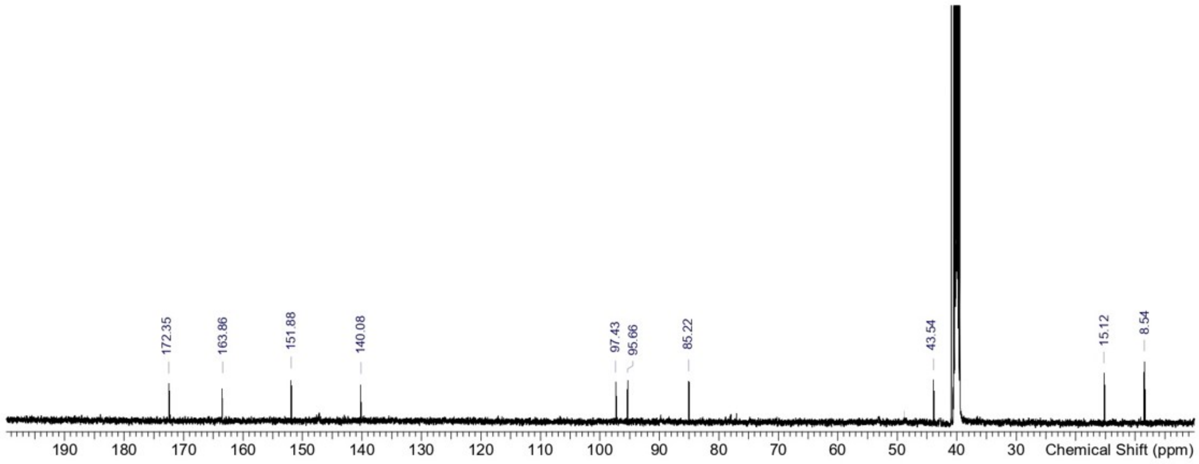

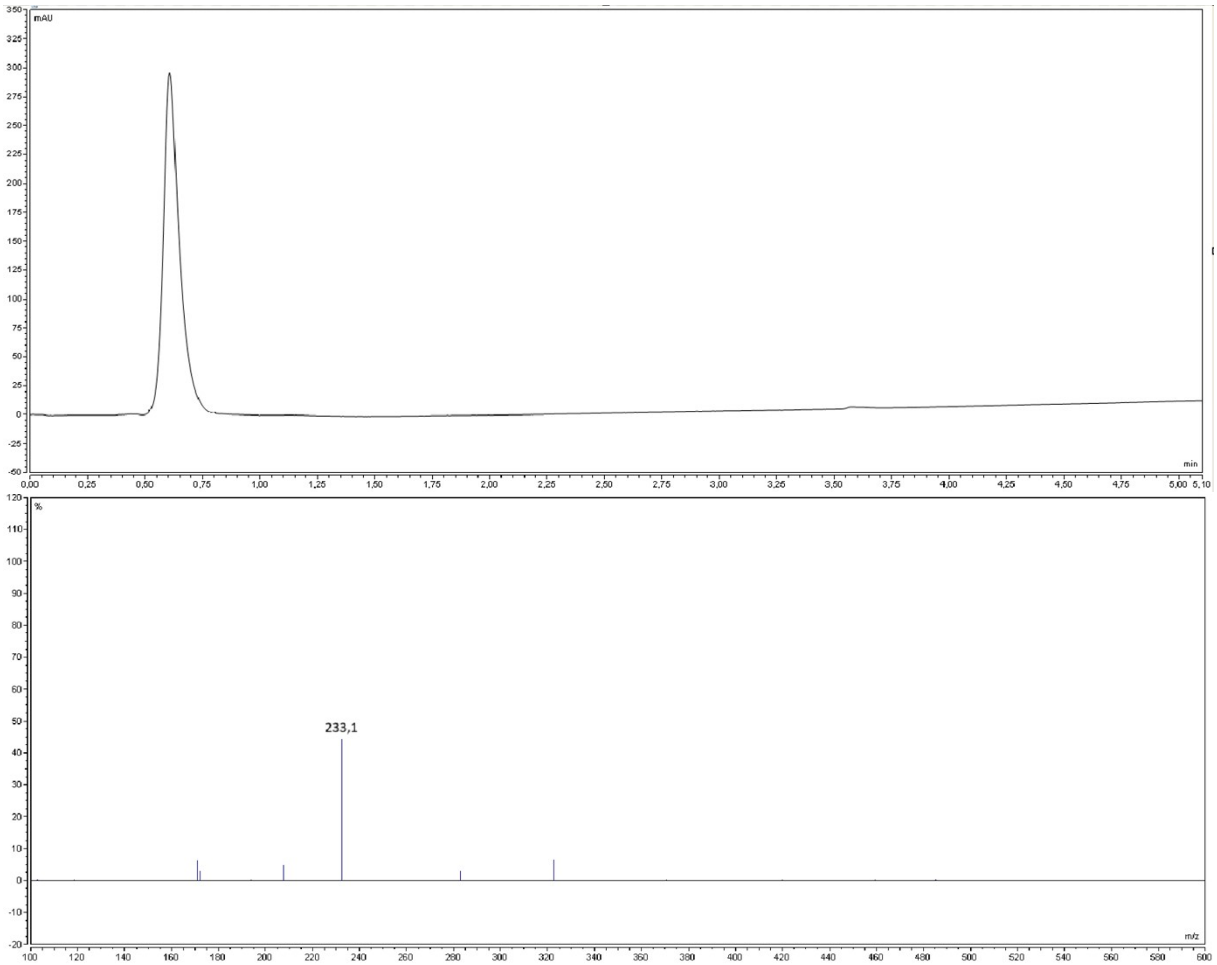

N-(3-(4-amino-2-oxopyrimidin-1(2H)-yl)propyl)cyclopropanecarboxamide (**15**)

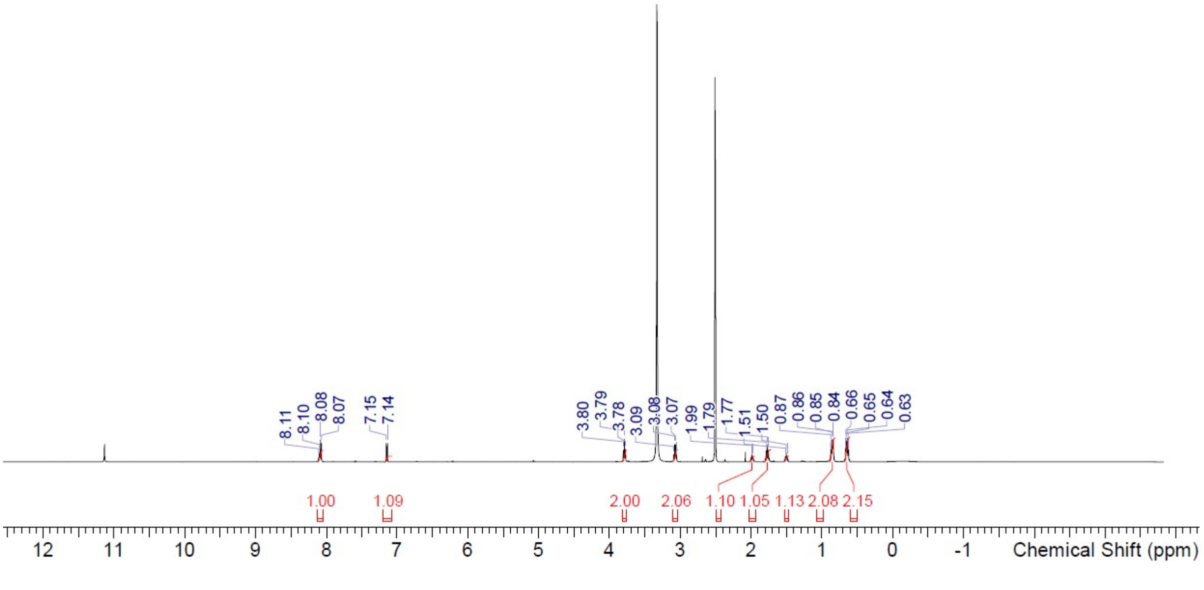

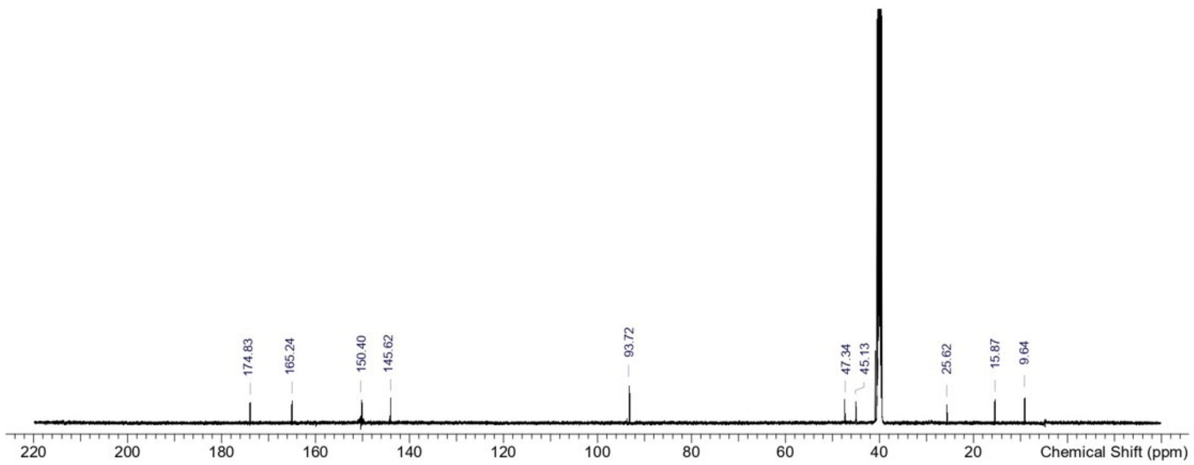

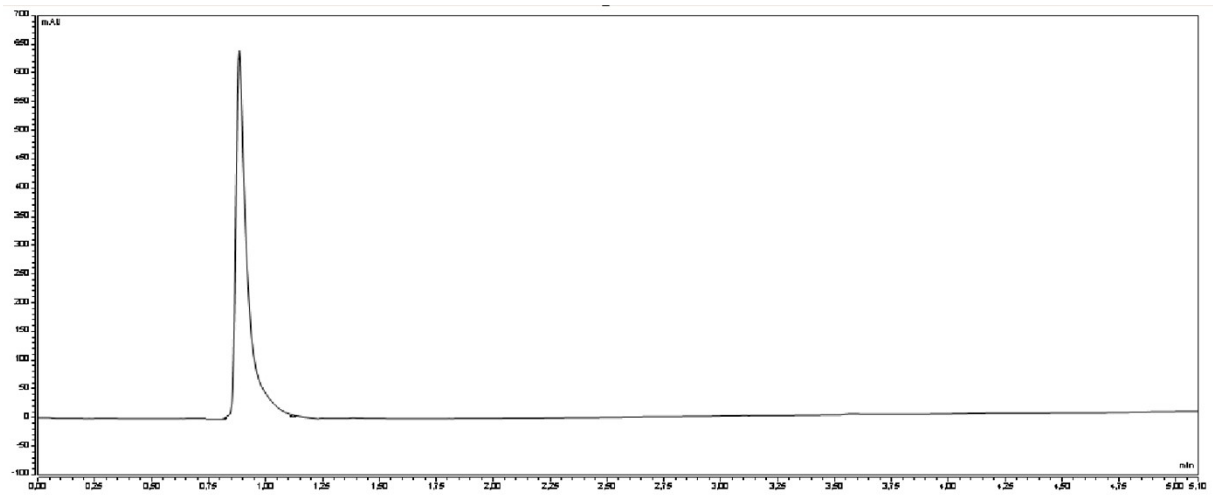

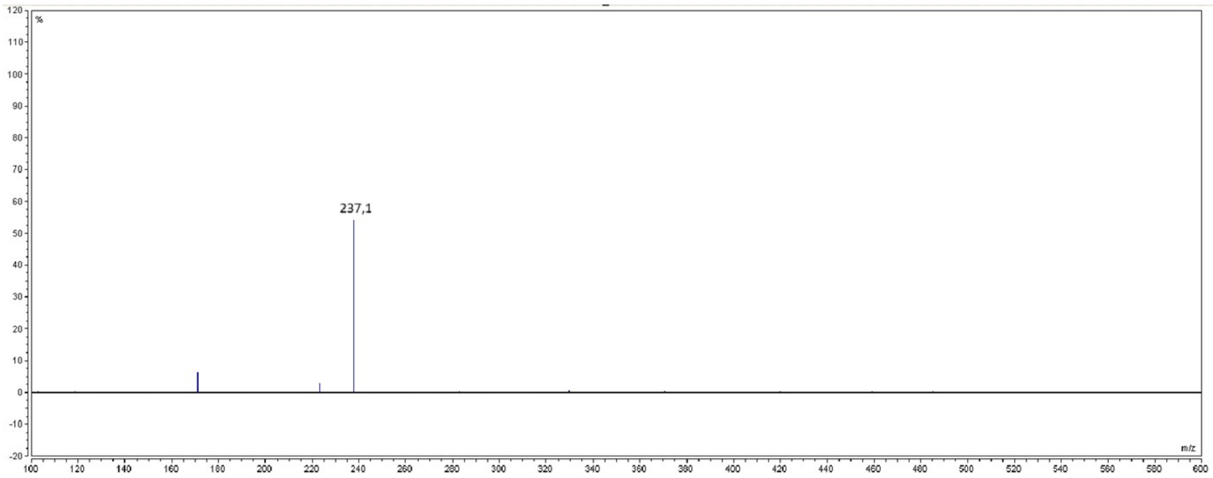

N-(3-(4-amino-2-oxo-1,2-dihydropyrimidin-5-yl)propyl)cyclopropanesulfonamide (**16**)

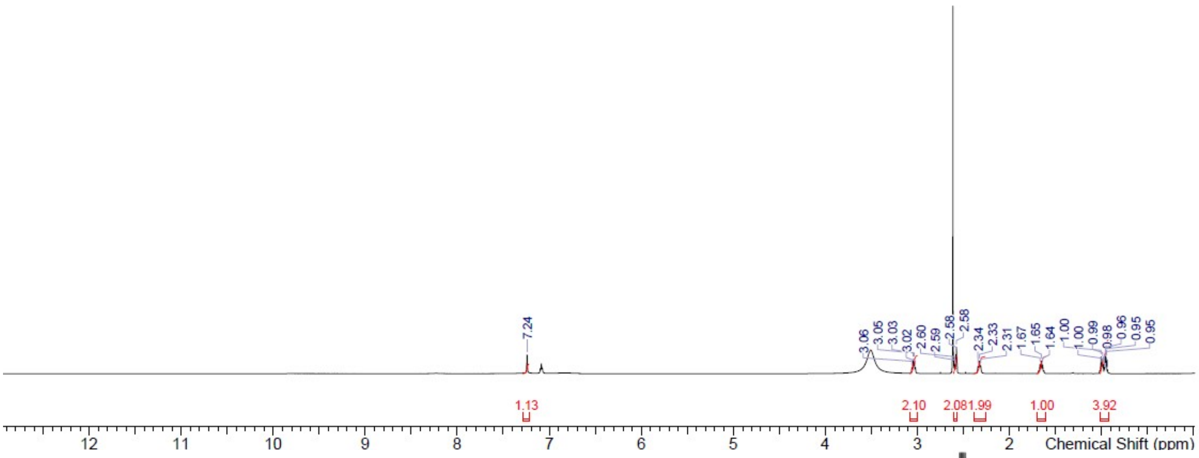
7.24
3.06
3.05
3.03
3.02
2.60
2.59
2.58
2.58
2.34
2.33
2.31
1.67
1.65
1.64
1.00
0.99
0.98
0.96
0.95
0.95
1.13
2.10
2.08
1.99
1.00
3.92
12
11
10
9
8
7
6
5
4
3
2
Chemical Shift (ppm)

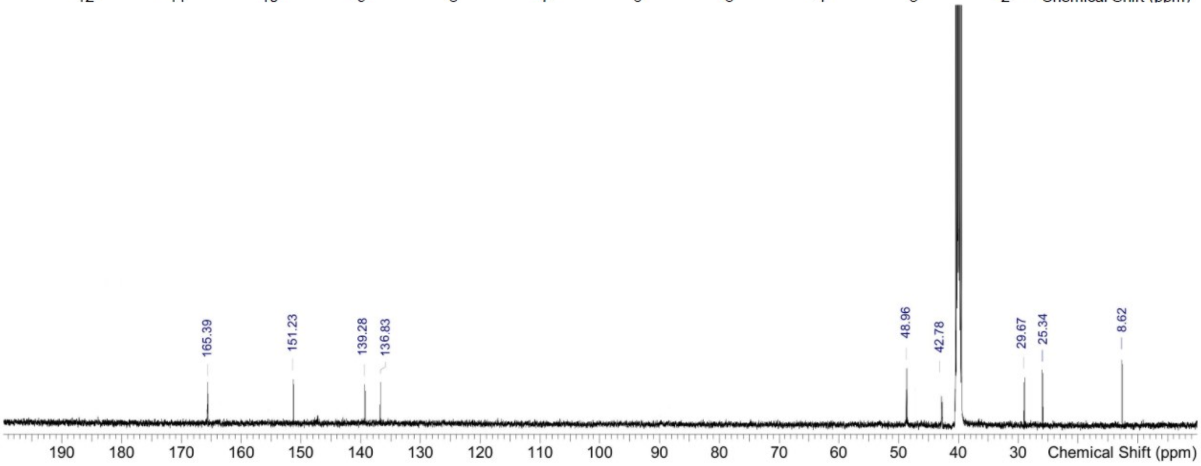
165.39
151.23
139.28
136.83
48.96
42.78
29.67
25.34
8.62
190
180
170
160
150
140
130
120
110
100
90
80
70
60
50
40
30
Chemical Shift (ppm)

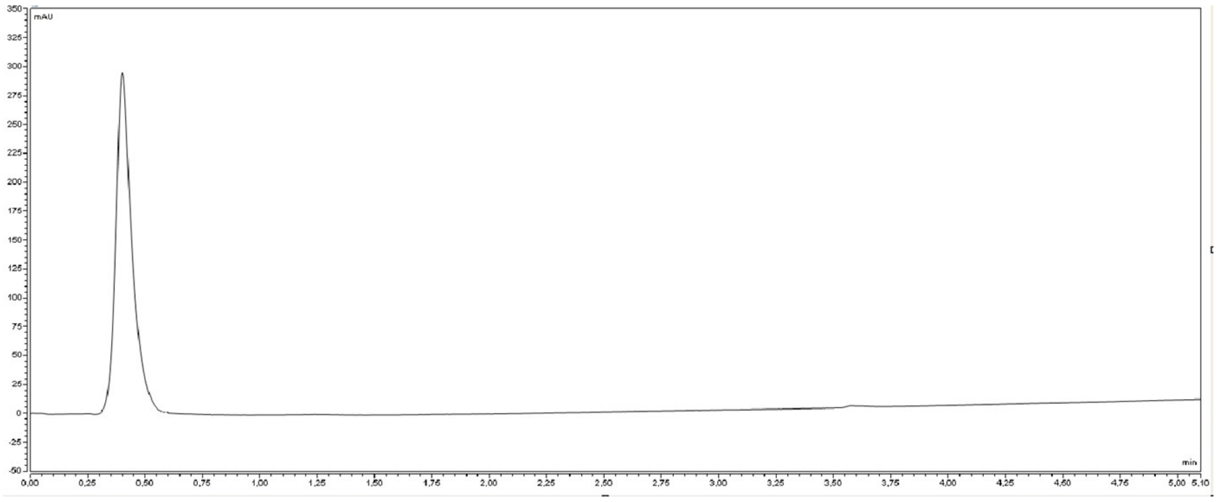
mAU
min

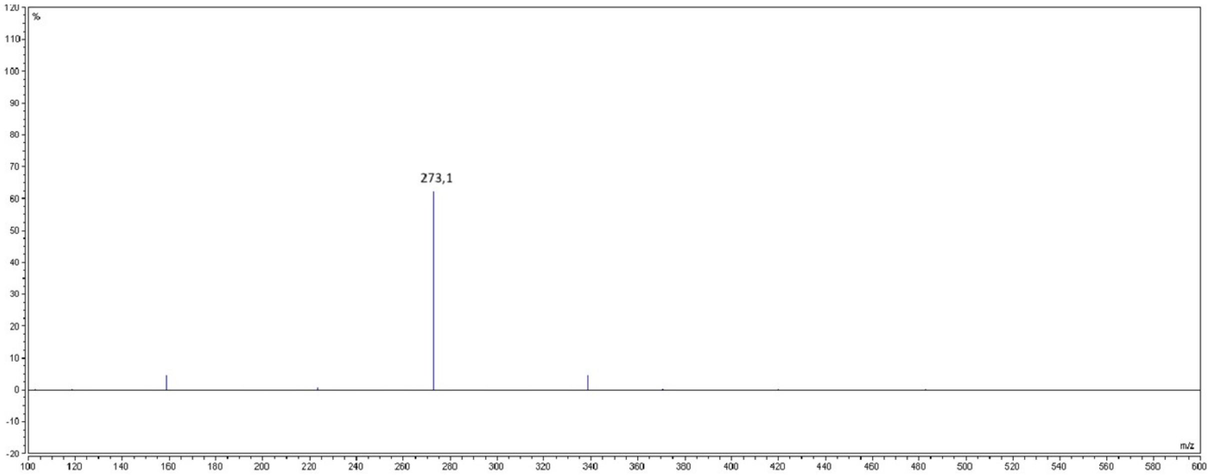

N-(3-(4-amino-2-oxo-1-(tetrahydrothiophen-2-yl)-1,2-dihydropyrimidin-5-yl)prop-2-yn-1yl)cyclopropanecarboxamide (**17**)

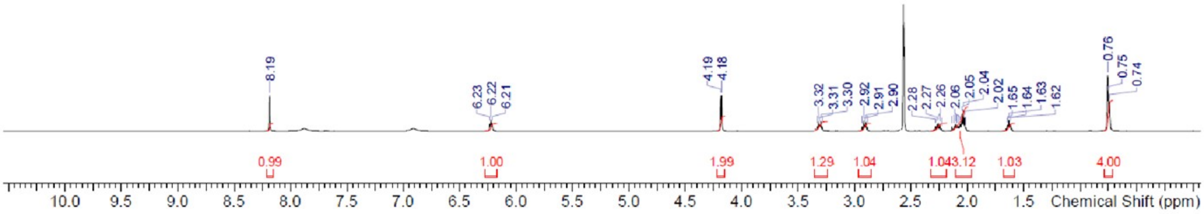

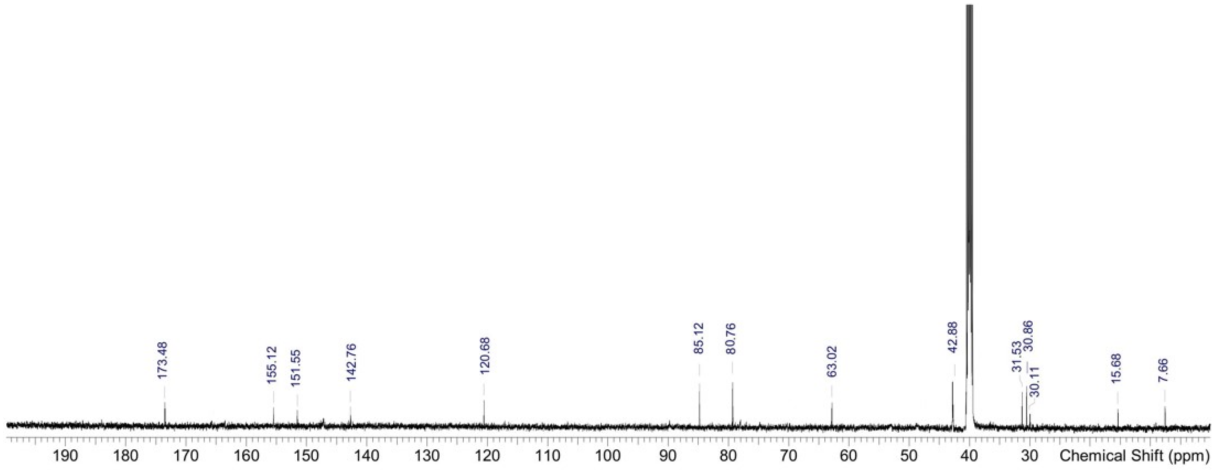

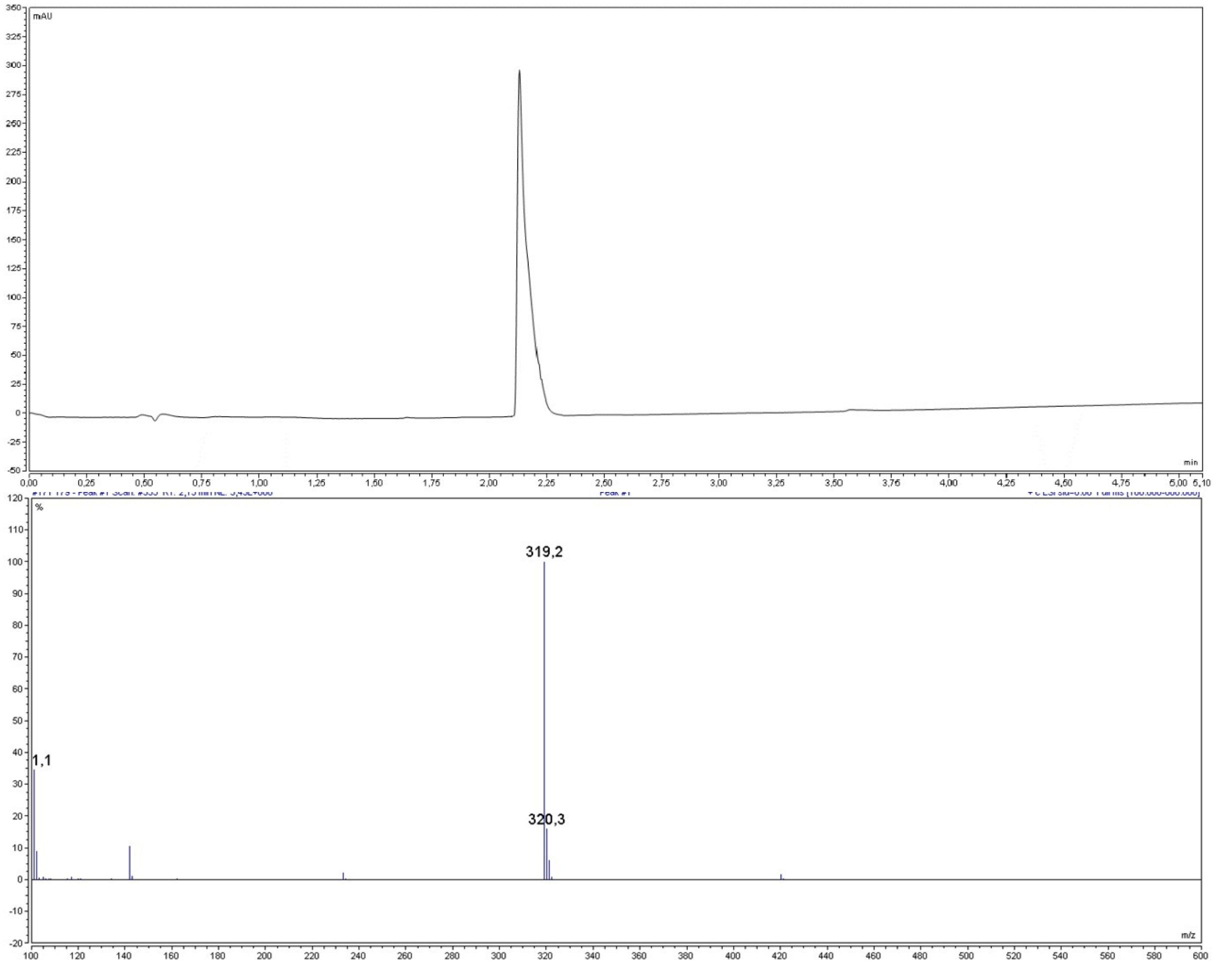

1. Kuzmič, P., Program DYNAFIT for the Analysis of Enzyme Kinetic Data: Application to HIV Proteinase. *Analytical Biochemistry* **1996,** *237* (2), 260-273.
2. Honold, A.; Lettl, C.; Schindele, F.; Illarionov, B.; Haas, R.; Witschel, M.; Bacher, A.; Fischer, M., Inhibitors of the Bifunctional 2-C-Methyl-d-erythritol 4-Phosphate Cytidylyl Transferase/2-C-Methyl-d-erythritol-2,4-cyclopyrophosphate Synthase (IspDF) of Helicobacter pylori. *Helvetica Chimica Acta* **2019,** *102* (3), e1800228.
3. Hamid, R.; Walsh, D. J.; Diamanti, E.; Aguilar, D.; Lacour, A.; Hamed, M. M.; Hirsch, A. K. H., IspE Kinase as an Anti-infective Target: Role of a Hydrophobic Pocket in Inhibitor Binding. *bioRxiv* **2024**, 2024.06.17.599194.
